# Wnt/β-catenin signaling pathway safeguards epigenetic stability and homeostasis of mouse embryonic stem cells

**DOI:** 10.1038/s41598-018-37442-5

**Published:** 2019-01-30

**Authors:** Ilda Theka, Francesco Sottile, Marco Cammisa, Sarah Bonnin, Marta Sanchez-Delgado, Umberto Di Vicino, Maria Victoria Neguembor, Karthik Arumugam, Francesco Aulicino, David Monk, Andrea Riccio, Maria Pia Cosma

**Affiliations:** 1 Centre for Genomic Regulation (CRG), The Barcelona Institute of Science and Technology, Dr. Aiguader 88, 08003 Barcelona, Spain; 2 Institute of Genetics and Biophysics ‘A. Buzzati-Traverso’, CNR, 80131 Naples, Italy; 3 Department of Environmental, Biological and Pharmaceutical Sciences and Technologies, Second University of Naples, 81100 Caserta, Italy; 4 Imprinting and Cancer Group, Cancer Epigenetic and Biology Program, Institut d’Investigació Biomedica de Bellvitge, Hospital Duran i Reynals, Barcelona, Spain; 5 Universitat Pompeu Fabra, (UPF), Dr. Aiguader 88, Barcelona, 08003 Spain; 6 Institució Catalana de Recerca i Estudis Avançat (ICREA), Pg. Lluís Companys 23, 08010 Barcelona, Spain

## Abstract

Mouse embryonic stem cells (mESCs) are pluripotent and can differentiate into cells belonging to the three germ layers of the embryo. However, mESC pluripotency and genome stability can be compromised in prolonged *in vitro* culture conditions. Several factors control mESC pluripotency, including Wnt/β-catenin signaling pathway, which is essential for mESC differentiation and proliferation. Here we show that the activity of the Wnt/β-catenin signaling pathway safeguards normal DNA methylation of mESCs. The activity of the pathway is progressively silenced during passages in culture and this results into a loss of the DNA methylation at many imprinting control regions (ICRs), loss of recruitment of chromatin repressors, and activation of retrotransposons, resulting into impaired mESC differentiation. Accordingly, sustained Wnt/β-catenin signaling maintains normal ICR methylation and mESC homeostasis and is a key regulator of genome stability.

## Introduction

The evolutionarily conserved Wnt/β-catenin signaling pathway controls many cellular and developmental processes, including cell proliferation, cell fate determination and tissue homeostasis^1^. Mutations affecting the Wnt/β-catenin pathway often lead to disease, cancer progression and developmental defects.

The canonical Wnt/β-catenin-dependent pathway integrates membrane, cytoplasmic and nuclear components, such as Wnt ligands, Frizzled (FZD) receptors and co-receptors, AXIN/glycogen synthase kinase 3 (GKS3)/Adenomatosis polyposis coli (APC)/Casein Kinase I (CKI) destruction complex, β-catenin protein and several transcription factors^1,2^. In the absence of Wnt ligands, cytoplasmic β-catenin is constantly degraded by the action of the AXIN/GSK3/APC/CKI destruction complex. On the contrary, the destruction complex is disassembled when Wnt ligands bind to the FZD receptors. As a consequence, β-catenin translocates to the nucleus where it associates with TCF/LEF (T-cell factor/lymphoid enhancing factor) nuclear complex and activates Wnt targeted gene expression^3^.

During embryogenesis Wnt/β-catenin signaling plays a fundamental role in the establishment of both dorso-ventral and anterior-posterior axis and its role is essential for normal gastrulation. Indeed, β-catenin knockout embryos are lethal since they fail to develop the mesodermal and endodermal germ layers^4,5^. Accordingly, Wnt/β-catenin represents a key pathway for mouse embryonic stem cell (mESC) identity and homeostasis.

Mouse ESCs, derived from the inner cell mass (ICM) of the blastocyst^6,7^ are pluripotent stem cells, which are able to generate the three germ layers and can be expanded *in vitro* indefinitely. Their long-term self-renewal ability has been attributed to the protein regulatory network that includes several pluripotency factors, such as *Nanog*, *Oct4* and *Rex1*, among others^8–11^. In this context, the role of β-catenin during mESC differentiation has been shown to be indispensable, since β-catenin null mESCs undergo apoptosis at the onset of the differentiation process^12–14^. However β-catenin function in ESC self-renewal has been largely debated^14–20^. The dual role of β-catenin can be attributed to its capacity to form complexes with many downstream factors, including key pluripotency genes, such as *Oct4*^21,22^.

In parallel to the core pluripotency factors, several epigenetic mechanisms control mESC identity through chromatin remodeling processes^23^. In particular, reversible changes on DNA methylation, followed by histone modifications, control both pluripotency and differentiation genes in mESCs, recapitulating the epigenetic profile of the pre-implantation embryo stage^24,25^. While developmental genes are characterized by flexible and reversible epigenetic regulation mechanisms to allow their dynamic expression, stable DNA methylation ensures silencing and protection of retrotransposons from moving around in the genome and causing potential mutations^26^. The same applies to imprinted genes, which reside in clusters^27^ and are regulated from one major *cis*-acting element called the imprinting control region, ICR. In mammals, DNA methylation is maintained stable and it can be propagated through cell division^28–30^ by a mechanism of DNA methylation maintenance coupled to DNA replication, which involves the action of different enzymes including DNA methyltransferase I (DNMT1). Along with DNA methylation, other epigenetic factors, such as ZFP57, KAP1, DNMT1, H3K9me3 and others, are involved in marking the ICRs and in protecting the methylated DNA. Indeed, loss of ZFP57, KAP1, DNMT1 or other repressors, leads to loss of imprinting and genomic instability in mESCs, and, thereby, to embryonic lethality^27,31–34^.

Epigenetic instability in imprinted genes and transposons has been observed in several mESC lines and can be attributed to culture conditions, sex of the cells, isolation procedures^35^ or prolonged *in vitro* culture of mESCs^36–39^. In particular, mESCs with global loss of methylation at the ICRs have been shown to contribute to chimeras, but mice developed several types of tumors by one year of age^40^. The mechanisms causing genomic aberrations and destabilization are still debated. However, downregulation of several epigenetic factors, such as DNMT1, KAP1, G9a, has been correlated with the epigenetic instability of the cells^34,41–46^.

Mouse embryonic stem cells represent an essential model to study *in vitro* the mechanisms that regulate embryo development. Therefore, it is important to fully understand the mechanisms that control cell identity, genomic stability and cell homeostasis. Wnt/β-catenin signaling has been investigated to be crucial for gene transcriptional regulation of mESCs, including pluripotency genes. Though, the connection between Wnt signaling and the epigenetic regulatory mechanisms has not been elucidated up to now. Here we investigated a novel role of Wnt/β-catenin signaling as a key player involved in epigenetic changes that preserve mESC identity and genome stability. We found that mESCs cultured *in vitro* for prolonged time showed loss of Wnt activity and downregulation of β-catenin protein, which correlated with a general loss of DNA methylation, affecting the ICRs, and leading to impaired mESC differentiation. On the contrary, sustained levels of Wnt/β-catenin ensure ICR methylation maintenance over time, suggesting a possible role for this signaling pathway in the protection of silent genomic regions and, therefore, in the maintenance of the genomic stability.

## Results

### Wnt/β-catenin activity is downregulated in mESCs after prolonged *in vitro* culture

The functional role of the Wnt/β-catenin pathway has been widely investigated in pluripotent stem cells. While the activation of Wnt pathway is indispensable for mouse embryonic stem cell (mESC) differentiation, its role in self-renewal and cell identity maintenance has been largely debated. Thus, we decided to analyze the activity of the Wnt/β-catenin pathway in mESCs cultured for a prolonged time, in particular its influence on pluripotency and homeostasis, including cell proliferation, differentiation potential and epigenetic stability.

**Figure 1:**
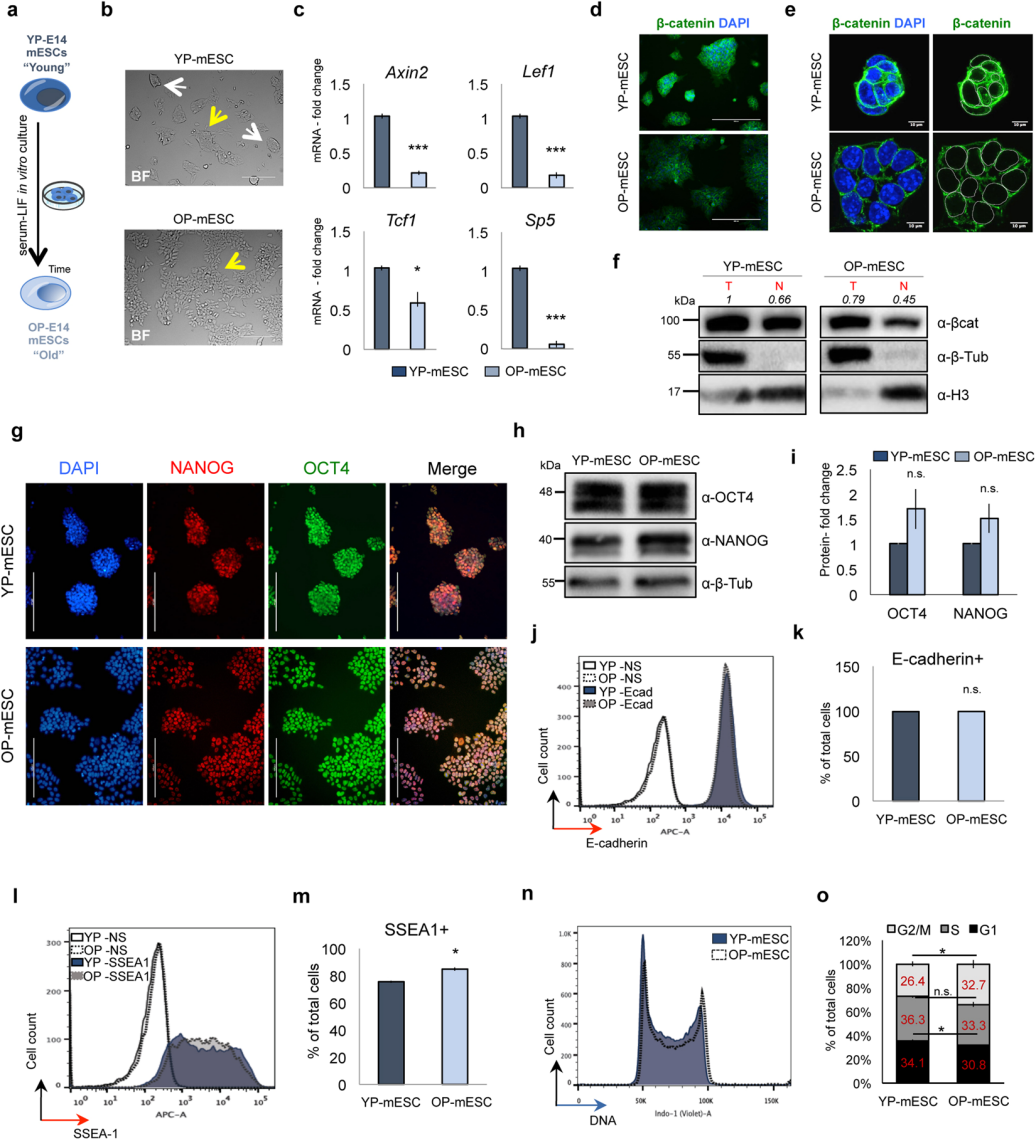
Prolonged *in vitro* cell culture of E14 mouse embryonic stem cells (mESCs) correlates with low Wnt/β-catenin activity. (**a**) Schematic representation of Young (YP) and Old passage (OP) E14 mESCs. (**b**) Representative bright field images of YP- and OP-mESCs. Round-shaped and flat colonies are indicated by white and yellow arrow, respectively. (**c**) Quantitative real-time PCR showing the expression profiles of *Axin2*, *Lef1*, *Tcf1*, *Sp5* in YP- and OP- mESCs. The transcriptional levels are normalized to *Gapdh* as reference gene. Data are represented as fold change (2^−ΔΔCt^) relative to the YP-E14 mESCs and means of n = 3 independent experiments ± SE. (**d**,**e**) Representative immunofluorescence (**d**) and confocal microphotographs (**e**) of β-catenin. Nuclear demarcation is indicated by white circles (right panel). (**f**) Western blot analysis showing total and nuclear β-catenin protein in YP- and OP-mESCs and its quantification (n = 1) relative to total β-catenin in YP-mESCs. For quantification, densitometric analysis was performed with ImageJ software. The quantification reflects the relative amounts as a ratio of each protein band relative to their loading control. (**g**) Representative immunofluorescence images showing OCT4 (green), NANOG (red) and their merge in YP- and OP-E14 mESCs. (**h**,**i**) Representative western blot analysis of OCT4 and NANOG in YP- and OP-mESCs (**h**) and its quantification represented as fold change over the protein amount in YP-mESCs and means of n = 3 independent experiments ± SE (**i**). (**f**,**h**,**i**) Full scan blots are available in Supplementary Fig. 6. (**j**–**m**) FACS-plot showing the percentage of E-cadherin +(**j**) and SSEA1 +cells (**l**) in YP- and OP- mESCs and its quantification (**k**,**m**) as means of 3 technical replicates ± SE (NS: non stained). (**n**,**o**) Representative cell cycle FACS profile analyzed with Flowjo software (**n**) and its quantification (**o**) represented as percentage of total cells and means of n = 3 independent experiments ± SE. Scale bar is 200 (**b**,**d**,**g**) and 10 μm (**e**). (**d**,**e** left panel, and **g**) Nuclei were stained with DAPI. (**f**,**h**) β-tubulin and H3 were used as loading controls. (**c**,**i**,**k**,**m**,**o**) Asterisks indicate statistical significance calculated by unpaired two-tailed t test analysis (n.s. not significant; *p-value < 0.05; ***p-value < 0.001).

To this aim we cultured E14 mESCs for several passages in the Serum + LIF medium. We observed that E14 mESCs cultured for many passages, around seventy, (old passage mESCs, henceforth called OP-mESCs), showed homogeneous morphology, characterized prevalently by flat clones, when compared to the same mESCs that were kept in culture for only fourteen passages (young passage mESCs, henceforth called YP-mESCs) (Fig. 1a,b). Similar results were obtained with GS1 mESCs, derived from a different strain, that were grown in prolonged culture conditions (around fifty passages, OP-mESCs) (Fig. S1a,b). In contrast, both E14 and GS1 YP-mESC cultures displayed heterogeneous morphology, including both round shaped and flat morphology clones (Figs 1b and S1b, upper panels). Pluripotent cell heterogeneity is due to fluctuation of pluripotency marker expression within the cell population. Even though cell heterogeneity can be found in almost all pluripotent stem cells, including induced pluripotent stem cells, the mechanisms causing gene expression variability and changes in morphology are still under investigation^47–53^.

Interestingly, both E14 and GS1 OP-mESCs showed low Wnt activity, since the Wnt targets *Axin2*, *Lef1*, *Tcf1* and *Sp5* were significantly downregulated, when compared to YP-mESCs (Figs 1c and S1c). In addition, total β-catenin protein was downregulated in E14 and GS1 OP-mESCs, as indicated by the microscope fluorescence intensity (Figs 1d–e and S1d,e). Additionally, by western blot analysis we observed lower amount of both total and nuclear β-catenin protein in OP-mESCs when compared to YP-mESCs (Figs 1f and S1f), suggesting again a reduction in the canonical Wnt/β-catenin signaling activity in OP-mESCs.

The difference in morphology of OP-mESCs did not correspond to significantly altered pluripotency gene expression. In particular, we compared the expression of NANOG and OCT4 protein levels among OP-mESCs and YP-mESCs for both E14 and GS1 strains (Figs 1g–i and S1g,i). We did not find any relevant difference in the expression pattern (Figs 1g and S1g) or significant changes in the protein level of NANOG and OCT4 (Figs 1h,i and S1h,i) among YP- and OP-mESCs. Thus, YP- and OP-mESCs expressed comparable levels of NANOG and OCT4.

Moreover, we performed FACS analysis to detect protein expression of the pluripotency cell membrane markers E-cadherin and SSEA1^54–59^. The expression of E-cadherin was similar between YP- and OP-mESCs in both cell lines (Figs 1j,k and S1j,k). The percentage of cells expressing SSEA1 was higher in OP-mESC E14 (Fig. 1l,m), but did not change in GS1 mESCs (Fig. S1l,m). These data further confirmed that the pluripotency genes were not downregulated after prolonged culturing or even they were slightly upregulated in OP cells, as in the case of E14 mESCs.

Since prolonged *in vitro* culturing^39,60^ and Wnt/β-catenin activity^61^ can affect cell proliferation, we compared cell cycle progression in both YP- and OP-mESCs. Both E14 and GS1 OP-mESCs displayed a significantly lower percentage of cells in G1 phase in comparison with the YP-mESCs (Figs 1n,o and S1n,o). Moreover, E14 OP-mESCs also showed a significant increase in the number of cells in G2/M phase, with respect to the YP-mESCs that on the contrary, displayed a higher number of cells in G1 and S phases (Fig. 1n,o). Overall, these data suggest that OP-mESCs are characterized by higher proliferation rate along with Wnt signaling down-regulation.

**Figure 2:**
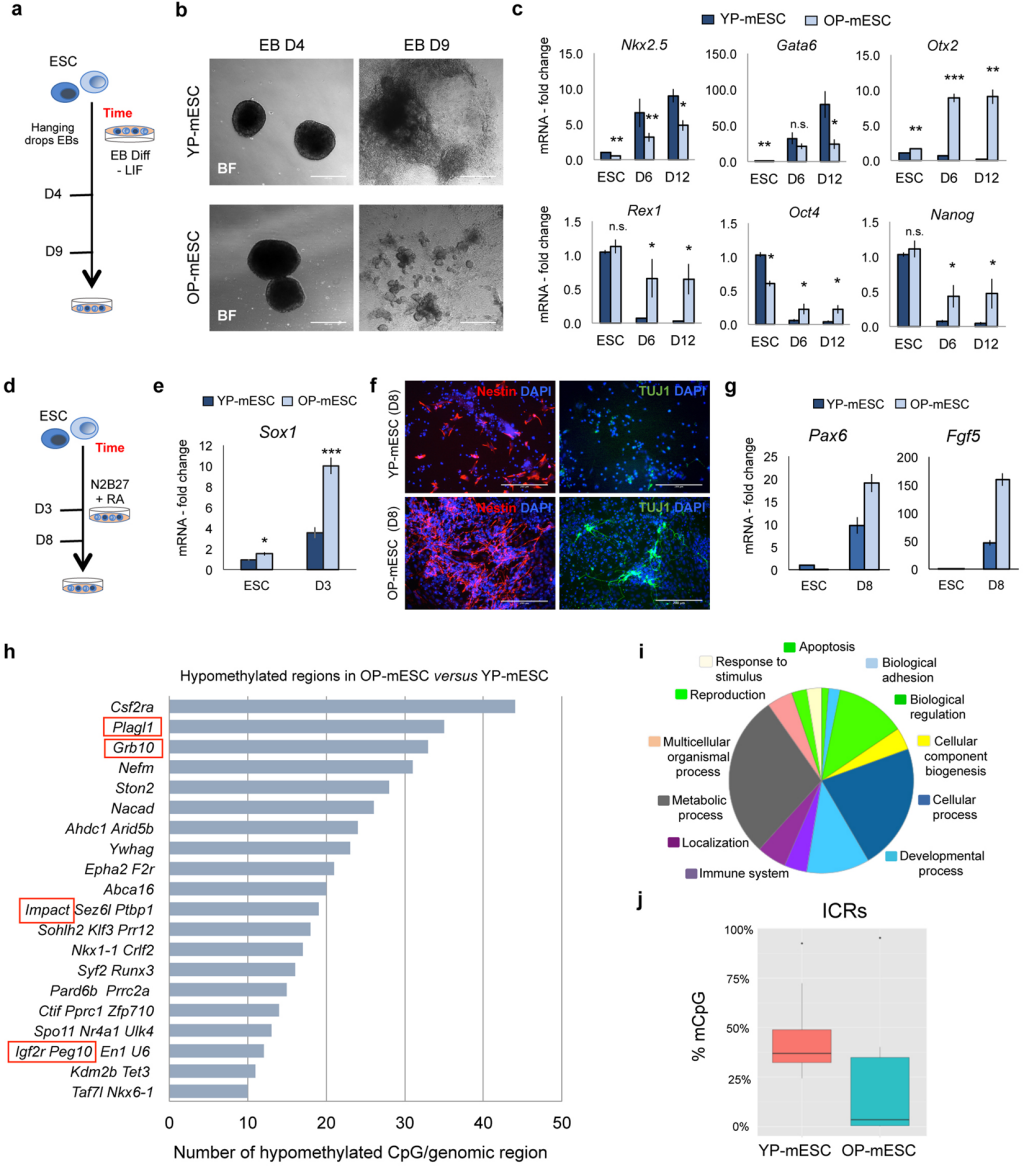
Old passage E14 mESCs show differentiation defects and loss of methylation at ICRs. (**a**) Schematic representation of embryoid body (EB) differentiation protocol of YP- and OP- mESCs. (**b**) Representative bright field images showing EBs at day 4 (D4) and 9 (D9) obtained from both YP- and OP- E14 mESCs. Scale bar is 400 μm. (**c**) Quantitative real-time PCR showing the expression profiles of differentiation genes (*Nkx2.5, Gata6, Otx2*) and pluripotency genes (*Rex1, Oct4, Nanog*) in YP- and OP- E14 mESCs (ESC) and during EB differentiation at day 6 (D6) and day 12 (D12). (**d**) Schematic representation of neural differentiation protocol of YP- and OP- mESCs. (**e**) Quantitative real-time PCR showing the expression profiles of *Sox1* at day 3 (D3) of N2B27 + retinoic acid (RA) treatment in YP- and OP-E14 mESCs (ESC). (**f**) Representative immunofluorescence images showing Nestin (left panels) and III β-tubulin (TUJ1, right panels) protein expression in YP- and OP- mESCs at day 8 (D8) of neural differentiation. (**g**) Quantitative real-time PCR experiment showing the expression profiles of *Pax6* and *Fgf5* at day 8 (D8) of N2B27 + retinoic acid (RA) treatment in YP- and OP- E14 mESCs (ESC). **(c**,**e**,**g**) The transcriptional levels are normalized to *Gapdh* as a reference gene. Data are represented as fold change (2^−ΔΔCt^) relative to the YP-E14 mESCs and the results are means of n = 3 independent experiments ± SE (**c**,**e**) and means of n = 3 technical replicated for SD (**g**). (**c**,**e**) Asterisks indicate statistical significance calculated by unpaired two-tailed t test analysis (n.s. not significant; *p < 0.05; **p < 0.01; ***p-value < 0.001). (**h**) Number of hypomethylated common CpGs in OP- versus YP- E14 mESCs analyzed by RRBS covered by at least 10 reads and showed at least 25% of methylation reduction. Red rectangle indicates imprinted regions. (**i**) Gene ontology of hypomethylated regions in OP-E14 mESCs analyzed by PANTHER (www.pantherdb.org). (**j**) Box-plot, from min–max values, showing the distribution of mCpG levels at ICRs in YP- and OP-E14 mESCs determined by RRBS analysis. The plots indicate the first quartile, median (black line) and third quartile. Data are obtained from the average of n = 2 biological replicates.

Wnt signaling and β-catenin protein are essential for differentiation and cell fate determination^12–14,20,62^. Thus, we induced embryoid body (EB) formation from both E14 and GS1 YP- and OP-mESCs (Figs 2a and S2a) to evaluate their differentiation capacity. EBs can recapitulate *in vitro* many aspects of cell differentiation that occur during early embryogenesis^63^. Interestingly, even though all the mESCs that we used could form normal aggregates in suspension (Figs 2b and S2b, left panels) only YP-mESCs were able to generate beating EBs (Movies S1, S2) and to form large three-dimensional multicellular structures (Figs 2b and S2b, right and upper panel). In contrast, OP-mESCs gave rise to small structures, and we did not observe any beating EBs (Movies S3, S4) up to day 9 (Figs 2b and S2b, right and lower panels). We further characterized the EBs by analyzing transcriptional levels of genes corresponding to the three germ layers at day 6 (D6) and 12 (D12) of differentiation. For this we used undifferentiated YP- and OP-mESCs as controls (ESC). In particular, EBs derived from E14 or GS1 OP-mESCs showed lower level of the mesodermal marker *Nkx2*.*5* already at day 6 with respect to the YP-mESCs, while the endoderm marker *Gata6* was significantly downregulated only at day 12 of EB differentiation (Figs 2c and S2c). These results were consistent with already published studies, which reported that Wnt/β-catenin pathway activity is essential for mesoendoderm specification^12,62^. On the other hand, the ectodermal marker *Otx2* was significantly upregulated in OP cells already at the mESC stage and it further increased with differentiation, being expressed at much higher level in OP-EBs with the respect to YP-EBs. This result suggests that the differentiation could be biased toward ectoderm (Figs 2c and S2c), which was confirmed when we induced neural differentiation^64,65^ of both YP- and OP-mESCs (Figs 2d and S2d). Indeed, OP-mESCs expressed higher levels of *Sox1*, when compared to YP-mESCs and this was also the case at day 3 of neural differentiation (Figs 2e and S2e). We confirmed these results by analyzing the expression of other neural markers at day 8 (D8) of differentiation, such as Nestin and III β –tubulin (TUJ1) and *Pax6* (Figs 2f–g and S2f,g). OP-mESCs expressed higher level of Nestin and III β –tubulin (TUJ1) protein at day 8 (D8) of neural differentiation, with respect to YP-mESCs, in both cell lines (Figs 2f and S2f). Moreover, both Nestin and III β –tubulin (TUJ1) positive cells obtained from OP-mESCs were characterized by a more branched and elongated morphology, suggesting a faster neural differentiation (Figs 2f and S2f). *Pax6* was upregulated in OP-mESC, in both E14 and GS1 (Figs 2g and S2g, left plot) and although *Fgf5* was upregulated in E14 OP- neural precursors, it showed lower levels in differentiated GS1 OP-mESCs compared to their YP counterpart (Figs 2g and S2g, right plot). Surprisingly, although similar levels of pluripotency genes were expressed in both YP- and OP-mESCs, during differentiation the levels of *Rex1*, *Oct4* and *Nanog*, remained much higher in OP-EBs with respect to the YP-EBs (Figs 2c and S2c, lower plots). These data suggest that the differentiation potential of mESCs, in particular toward the meso-endodermal germ layers, is strongly impaired in prolonged culture condition, and correlates with high level of pluripotency genes in both OP-mESCs and OP-EBs.

### Reduced activity of Wnt/β-catenin signaling in OP-mESCs correlates with loss of DNA methylation, affecting also ICRs

Several epigenetic alterations causing DNA methylation changes have been associated with prolonged *in vitro* cell culture^36^. In pluripotent stem cells, DNA methylation regulates many cellular processes including cell differentiation. Altered DNA methylation has often been associated with impaired differentiation capacity of mESCs^44^. In this context, Wnt/β-catenin pathway regulates pluripotent stem cell differentiation, however its involvement in DNA methylation has not been explored. We, therefore, wondered whether OP-mESCs, which express low level of β-catenin protein and Wnt target transcripts, could show any change in DNA methylation levels. Thus, we performed Reduced Representation Bisulfite Sequencing (RRBS), a technique that combines restriction enzymes and bisulfite sequencing to enrich for the genomic areas with high CpG content^66^, using both YP- and OP-mESCs. Only 3 genomic regions, localized close to *Repin1*, *Nkx2-1* and *Gm11846* genes, were hypermethylated in E14 OP-mESCs after prolonged culture, when compared to the YP-mESCs (Table S1). In contrast, we found a general hypomethylation in E14 OP-mESCs. Indeed, about 100 genomic CpG-rich regions displayed reduced DNA methylation in OP-mESCs with respect to YP-mESCs (Fig. 2h and Table S1). The coding genes nearby these hypomethylated genomic regions belong to different gene families and they control several biological processes, including metabolic and developmental processes, as analyzed by PANTHER functional classification (Fig. 2i). Among the list of hypomethylated regions we found several imprinted genes. Imprinted genes have been previously described to be crucial for metabolic and developmental process regulation^67,68^. In particular, the ICRs corresponding to *Plagl1*, *Grb10*, *Impact*, *Igf2r* and *Peg10* loci showed reduced DNA methylation in many CpGs in OP-mESCs but not in YP-mESCs (Fig. 2h). ICRs control many elements within the imprinted clusters and they have been shown to be generally stable in the pre-implantation embryo, and to be methylated in only one allele^27,32^. By analyzing only ICRs, we observed that YP-mESCs showed a normal profile of DNA methylation (around 50%, corresponding to one specific allele), while methylation was lost in several ICRs in OP-mESCs (Fig. 2j). Our results indicate that impairment of the ICR status, such as loss of DNA methylation, can affect pluripotency and differentiation potential of mESCs^40^.

To validate the RRBS data and to examine the methylation profile of additional ICRs, we performed Combined Bisulfite Restriction Analysis (COBRA) coupled to pyrosequencing analysis. Methylation analysis by COBRA includes the use of methylation sensitive restriction enzymes that can digest DNA only when methylated. As expected, some of the ICRs were hypomethylated in OP-mESCs and not in YP-mESCs, such as *Airn*, *Rasgrf1*, *Peg10* and *Grb10*, as shown by the enzymatic digestion pattern (Fig. S2h). On the contrary *Ig-DMR* and *Gnas XL* did not show differences in DNA methylation (Fig. S2h). In parallel, we also included genomic DNA extracted from a wild type mouse (Ctrl gDNA) as a positive control, carrying a normal methylation profile, and from Zfp57 knockout mESCs (Zfp57 KO) that have been shown to lose methylation at several ICRs^34^.

Finally, to quantify the DNA methylation changes observed by COBRA, we performed pyrosequencing analysis. Even in this case, E14 YP-mESCs showed normal levels of DNA methylation (around 50%) at the analyzed ICRs (*Airn*, *Rasgrf1*, *Peg10*, *Grb10*, *Ig-DMR* and *Gnas XL*) that was comparable to the mouse genomic DNA (Ctrl gDNA) (Fig. S2i). On the contrary, OP-mESC methylation profile was similar with that of Zfp57 KO mESCs, as *Airn*, *Rasgrf1*, *Peg10* and *Grb10* had less than 20–30% of methylation. However, no changes were detected with passages in *Ig-DMR* and *Gnas XL* ICRs (Fig. S2i). In addition, by performing bisulfite-PCR sequencing we also observed loss of methylation at several CG dinucleotides in both *KvDMR* (also called *Kcnq1*) and *Inpp5fV2* ICRs in E14 OP- but not in YP-mESCs (Fig. S2j). The *KvDMR* ICR was hypomethylated also in the GS1 OP-mESCs when compared to YP-mESCs (Fig. S2j,k left). However, the *Inpp5fV2* ICR was already hypomethylated in the GS1 YP-mESCs and did not show any further loss of methylation with passages (Fig. S2k, right).

Overall these data indicate that downregulation of endogenous Wnt signaling and of β-catenin protein level occurs in prolonged mESC cultures and this correlates with loss of DNA methylation. In particular, we observed loss of methylation at several ICRs, including both maternally and paternally methylated loci that, in turn, control the expression of a variety of coding and non-coding genes within the imprinted clusters.

### Wnt/β-catenin activity “protects” the ICRs against de-methylation

**Figure 3:**
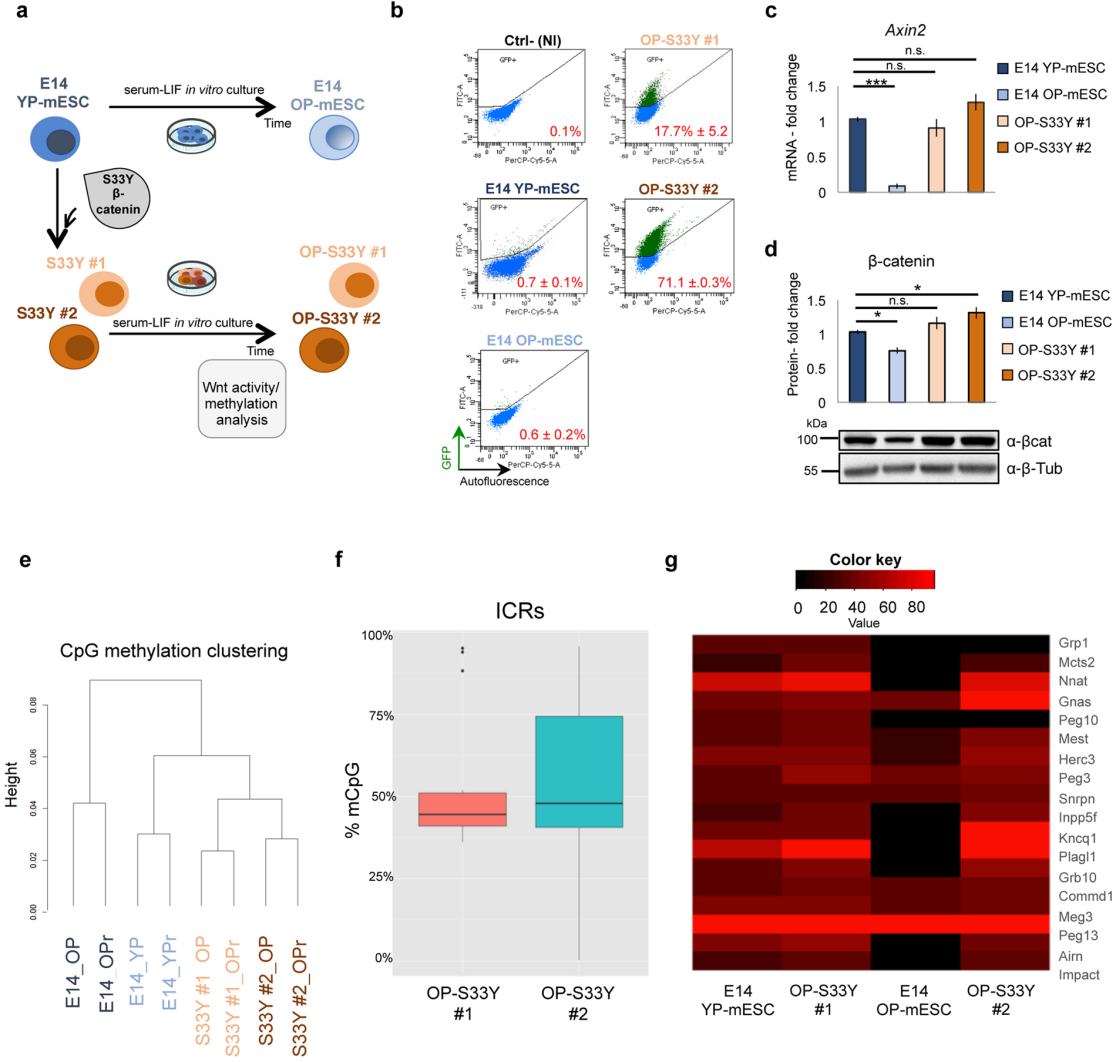
β-catenin overexpressing mESC clones maintain high Wnt/β-catenin activity and normal ICR methylation level after several *in vitro* passages. (**a**) Scheme showing how β-catenin overexpressing mESC clones (S33Y #1, #2) were obtained and grown. (**b**) Representative FACS-plot showing the percentage of positive mESCs for 7TGP topflash reporter activity in YP-mESCs, OP-mESCs, OP-S33Y #1 and OP-S33Y #2 mESC clones. The non infected (NI) cells were used as negative control (Ctrl-). The FITC and the Per-CP-Cy5.5-A detectors were used to identify GFP +(y axis) and autofluorescence (false positive) cells (x axis). The number of recorded events spans from 12000 (OP-mESC) to 18000 (YP-mESCs). Data are represented as means of n = 3 independent experiments ± SD. (**c**) Quantitative real-time PCR experiments showing the expression profiles of *Axin2* in YP-mESCs, OP-mESCs, OP-S33Y #1 and OP-S33Y #2 mESC clones. The transcriptional levels are normalized to *Gapdh* as a reference gene. Data are represented as fold change (2^−ΔΔCt^) relative to the YP-E14 mESCs and the results are means of n = 3 independent experiments ± SE. Asterisks indicate statistical significance calculated by unpaired two-tailed t test analysis (n.s. not significant; ***p < 0.001). (**d**) Western blot analysis showing total β-catenin protein levels in E14 YP-mESCs, OP-mESCs, OP-S33Y #1 and OP-S33Y #2 mESC clones and its quantification. Data are represented as fold change over the protein amount in YP-mESCs and means of n = 3 independent experiments ± SE. β-tubulin was used as loading control. For western-blot quantification densitometric analysis was carried out by using ImageJ software. The quantification reflects the relative amounts as a ratio of each protein band relative to their loading control. (**e**) Cluster analysis of the four different mESCs. For each line 2 different biological replicates were represented. (**f**) Box-plot, from min–max values, showing the distribution of mCpG levels at ICRs in OP-S33Y #1 and OP-S33Y #2 mESC clones determined by RRBS analysis. The plots indicate the first quartile, median (black line) and third quartile. Data are obtained from the average of n = 2 biological replicates. (**g**) Heat-map representation of ICR methylation levels in YP-mESCs, OP-mESCs, OP-S33Y #1 and OP-S33Y #2 mESC clones.

To further investigate the implication of Wnt/β-catenin signaling in the control and protection of the ICRs against de-methylation, we generated two Wnt signaling mutant mESC clones overexpressing the S33Y-mutated β-catenin protein in E14 YP-mESCs. This β-catenin protein mutant is stable since it is not recognized and degraded by the AXIN/GSK3β/APC/CKI destruction complex and it is retained in the nucleus^69,70^. We named the two E14-derived mESC clones, carrying the S33Y-mutant β-catenin protein, S33Y-β-cat #1 and S33Y-β-cat #2. To test if sustained Wnt/β-catenin signaling activity could preserve methylation at ICRs, we cultured the clones in the conditions that were used to derive E14 OP-mESCs (Fig. 3a). We next tested the level of Wnt signaling by analyzing the activity of the topflash reporter (7TGP)^71^ in the old passage ESC clones (OP-S33Y-β-cat #1 and OP-S33Y-β-cat #2) and we compared them with both E14 YP- and OP-mESCs. OP-S33Y-β-cat #1 and OP-S33Y-β-cat #2 retained high level of Wnt activity as indicated by the top-flash reporter (Fig. 3b) and by high expression of *Axin2* (Fig. 3c). Since the 7TGP lentiviral vector carries a puromycin resistance cassette, prior to the Wnt activity analysis we selected the infected cells to ensure homogeneous expression of the reporter within the cell population in each condition. In parallel, to test the topflash reporter reliability we treated E14 mESCs with either DMSO or CHIR (3 μM) for 24 hours as previously reported^15,72^. As expected, the 7TGP reporter was activated upon CHIR but not DMSO treatment (Fig. S3a). Moreover, total β-catenin protein was maintained at similar level as in E14 YP-mESCs, rather than being downregulated as in E14 OP-mESCs (Fig. 3d).

Next, we tested the methylation status of OP-S33Y-β-cat #1 and OP-S33Y-β-cat #2 mutant clones by performing RRBS analysis and compared the mutant clones with both E14 YP- and OP-mESCs. The clusters and PCA analysis grouped together E14 YP-mESCs, OP-S33Y-β-cat #1 and OP-S33Y-β-cat #2 (Figs 3e and S3b), but not E14 OP-mESCs, which clusterized apart. In addition, the two replicates belonging to each sample perfectly correlated between them (Fig. S3c). As expected, OP-S33Y-β-cat #1 and OP-S33Y-β-cat #2 clones maintained the methylation at the ICRs at around 50%, indicating allele-specific methylation (Fig. 3f). Moreover, by plotting together all the conditions it was clear that E14 OP-mESCs showed loss of methylation at many ICRs if compared to YP-mESCs and to the mutant clones (Fig. 3g).

To further validate the RRBS data and to analyze additional ICRs, we performed COBRA analysis and pyrosequencing quantification (Fig. S3d,e). Methylation at *Airn*, *Rasgrf1*, *Grb10* and *Ig-DMR* ICRs was maintained normal, at around 50%. However, in the OP-S33Y-β-cat #2 clone *Gnas XL* and *Peg10* were hypermethylated and hypomethylated respectively, suggesting other possible and unpredictable cellular mechanisms, or perturbations occurring in specific clones that could be induced by selection effects. Finally, OP-S33Y-β-cat #1 and OP-S33Y-β-cat #2 mESC clones maintained a round shaped morphology also after several passages *in vitro* (Fig. S3f).

Overall these data indicate that sustained Wnt/β-catenin activity in mESCs cultured for several passages in Serum + LIF medium prevents the loss of methylation at ICRs and at other genomic regions. Accordingly, loss of Wnt activity can affect DNA methylation and, as a consequence, mESC differentiation potential.

### DNA hypomethylation results in loss of chromatin repressor recruitment at the ICRs

**Figure 4:**
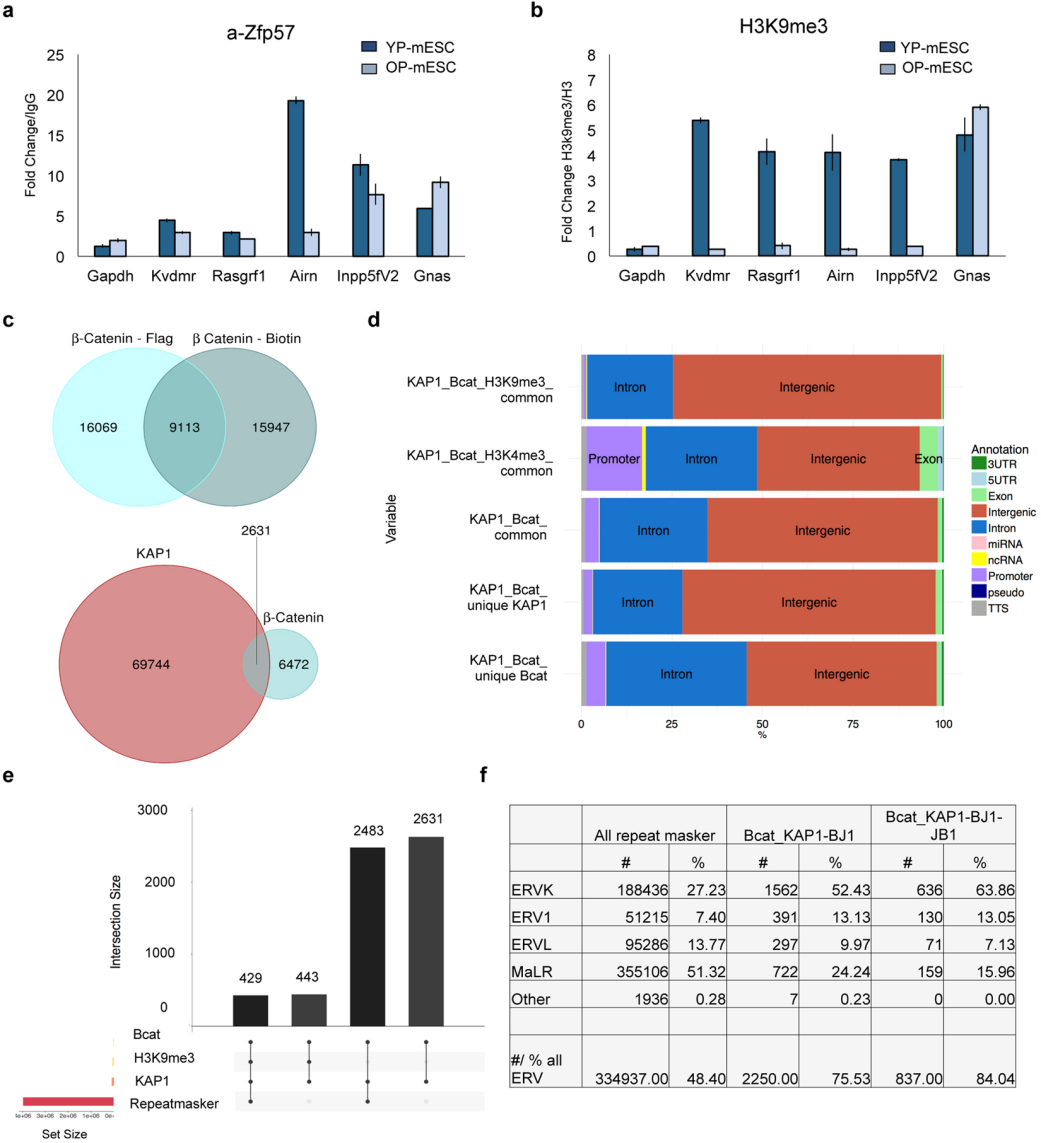
β-catenin and KAP1 share intergenic common DNA binding sites localized mainly on LTRs and ERVs. (**a**,**b**) Representative ChIP-qPCR experiment (out of n = 2 independent experiments) of ZFP57 (**a**) and H3K9me3 (**b**) recruitment at several ICRs. The data are represented as fold change (2^−ΔΔCt^) over IgG (**a**) or H3 (**b**) and means ± SD. (**c**) Venn diagram showing overlapping regions between ChIP-sequencing profiles of β-catenin and KAP1. The peaks between Flag- (β-catenin-Flag) and Biotin- (β-catenin-Biotin) tagged endogenous β-catenin published by Zhang and colleagues^73^ were intersected among them. The common bound regions were then overlapped with KAP1 ChIP-sequencing peaks performed in BJ1 mESCs by Anvar and colleagues^31^. (**d**) Bar chart showing genomic distribution of unique and common peaks among β-catenin, KAP1, H3K9me3, H3K4me3. (**e**) Bar chart showing ChIP-sequencing peaks intersection among KAP1, β-catenin, KAP1, H3K9me3 and Repeat masker. The number of common overlapping peaks is indicated on the top of the bars. (**f**) Table showing the different LTR and ERV families represented as number (#) and percentage (%) over Repeat maskers (column 2, 3), the total number of common overlapping peaks between β-catenin and KAP1 in BJ1 (column 4, 5), the total number of common overlapping peaks among β-catenin, KAP1 in BJ1 and KAP1 in JB1 mESCs (columns 6, 7).

To protect the methylated allele from de-methylation, several chromatin repressors are recruited at the ICRs. In particular, ZFP57 is highly expressed in mESCs and can directly bind to a methylated hexanucleotide DNA motif within imprinted control regions^34^. After binding, ZFP57 recruits KAP1, which in turn interacts with other heterochromatin-associated histone marks, including H3K9me3^33,34^. Thus, we decided to investigate whether ZFP57 and H3K9me3 levels changed during mESC passages along with the loss of DNA methylation at the ICRs. To this aim, we performed chromatin immunoprecipitation (ChIP)-qPCR experiments for both ZFP57 and H3K9me3 on some known ICR target regions^34^. Both ZFP57 and H3K9me3 binding decreased in *KvDMR*, *Rasgrf1*, *Airn* and *Inpp5fV2* ICRs, reflecting their methylation status. On the contrary, no reduction of ZFP57 and H3K9me3 binding occurs at the *Gnas* ICR, which did not show loss of DNA methylation in OP-mESCs (Figs 4a,b and S2h,i). *Gapdh* was used as negative control region and showed low levels of enrichment across all conditions as expected (Fig. 4a,b). Similar results were obtained in GS1 mESCs. GS1 OP-mESCs were characterized by loss of ZFP57 and H3K9me3 recruitment at *KvDMR*, *Rasgrf1*, *Airn*, but not at *Inpp5fV2*, which was already de-methylated in GS1 YP-mESCs (Figs S4a,b and S2k). These data show that ZFP57 and H3K9me3 ChIP levels are decreased at hypomethylated ICRs, consistently with the methylation profiles shown previously (Figs 2h–j, 3g and S2h,k).

### β-catenin and KAP1 proteins share common genomic bound regions

Having observed the effect of the Wnt/β-catenin signaling downregulation, we wondered whether β-catenin could play a direct or indirect role in the control of the ICR methylation and epigenetic changes. By analyzing published ChIP-sequencing datasets we observed that several β-catenin binding sites localized close to KAP1, which is responsible for the recruitment of repressors in silent genomic regions. In particular, we compared published β-catenin ChIP-sequencing profiles^73^ with those of KAP1^31^. We first overlapped ChIP-sequencing profiles between Biotin-tagged and Flag-tagged β-catenin, and we found 9113 common peaks (almost 69% of all peaks) (Fig. 4c, upper diagram), confirming the previously published results^73^. Next, we intersected these 9113 peaks with KAP1 ChIP-sequencing profiles^31^ carried out using two different mESC strains (BJ1 and JB1 mESCs) and we found 2631 common overlapping regions between β-catenin and KAP1 in BJ1 mESCs (Fig. 4c, lower diagram). Since the number of total bound regions was much lower in KAP1 ChIP-sequencing dataset in JB1 mESCs (38713 versus 72535 for KAP1 in BJ1 mESCs), we found less common regions bound by both β-catenin and KAP1 (Table S2). As expected, most of the common regions bound from both β-catenin and KAP1, were intergenic or within introns, around 64% and 30% respectively considering both KAP1 ChIP-sequencing data sets (KAP1 in BJ1 and JB1 mESCs) (Fig. 4d and Table S2).

In parallel, we observed that, among the overlapping peaks, around 400–500 regions were also enriched for either H3K9me3 or H3K4me3, which are associated to silent or active chromatin, respectively (Fig. S4c,d and Table S2). For these analysis, we used the already published ENCODE ChIP-sequencing data for H3K9me3 (GSM1000147) and H3K4me3 (GSM769008)^74^. As expected, the H3K9me3 peaks were located mostly within intergenic genomic regions, while the H3K4me3 bound sites were distributed among intergenic, introns and promoters, including CpG islands (Figs 4d, S4e and Table S2). Nevertheless, in the overlapping regions among β-catenin, KAP1 and H3K9me3 we could find only two ICRs (*Grb10* and *Meg3*), as potential targets of β-catenin. Notably, the same two ICRs were also bound by ZPF57^31^, which specifically binds to methylated ICRs, as we previously observed in the overlapping regions between β-catenin and ZFP57. However, in this case, the total number of common binding sites was very low (Table S2).

Interestingly, almost all common genomic regions, bound by β-catenin and KAP1 were enriched in repeats (94% and 97% considering KAP1 datasets carried out in BJ1 and JB1 mESCs, respectively). This was also true when we overlapped the data with H3K9me3 peaks (Fig. 4e and Table S2). Among the different repeats the most enriched were the LTRs, which constitute 40% of the common regions bound by β-catenin and KAP1 (Fig. S4f and Table S3). Accordingly, 78–79% of the LTRs bound by both β-catenin and KAP1 were located within intergenic regions (Fig. S4g), confirming the previous data, showed in Fig. 4d and Supplementary Table S2.

Long-terminal repeat (LTR) elements belong to the third repeat class and represent 10% of all mammalian transposable elements^75,76^. All mammalian LTRs derive from the vertebrate-specific endogenous retroviral elements (ERVs), which can be grouped into sub-classes (I–III) and subfamilies, such as murine retroviral-related sequences (MURRSs, class I), ERVK, class II, ERVL and mammalian apparent LTR retrotransposons (MaLR, (class III), and others^75–77^. The expression of retroelements has been previously detected in different developmental stages. However, in most tissues and during embryo development the transcription of ERVs is counteracted by several epigenetic mechanisms, including DNA methylation, chromatin repressors (i.e. KAP1) and heterochromatin-associated histone marks, such as H3K9me3^46,78–80^.

**Figure 5:**
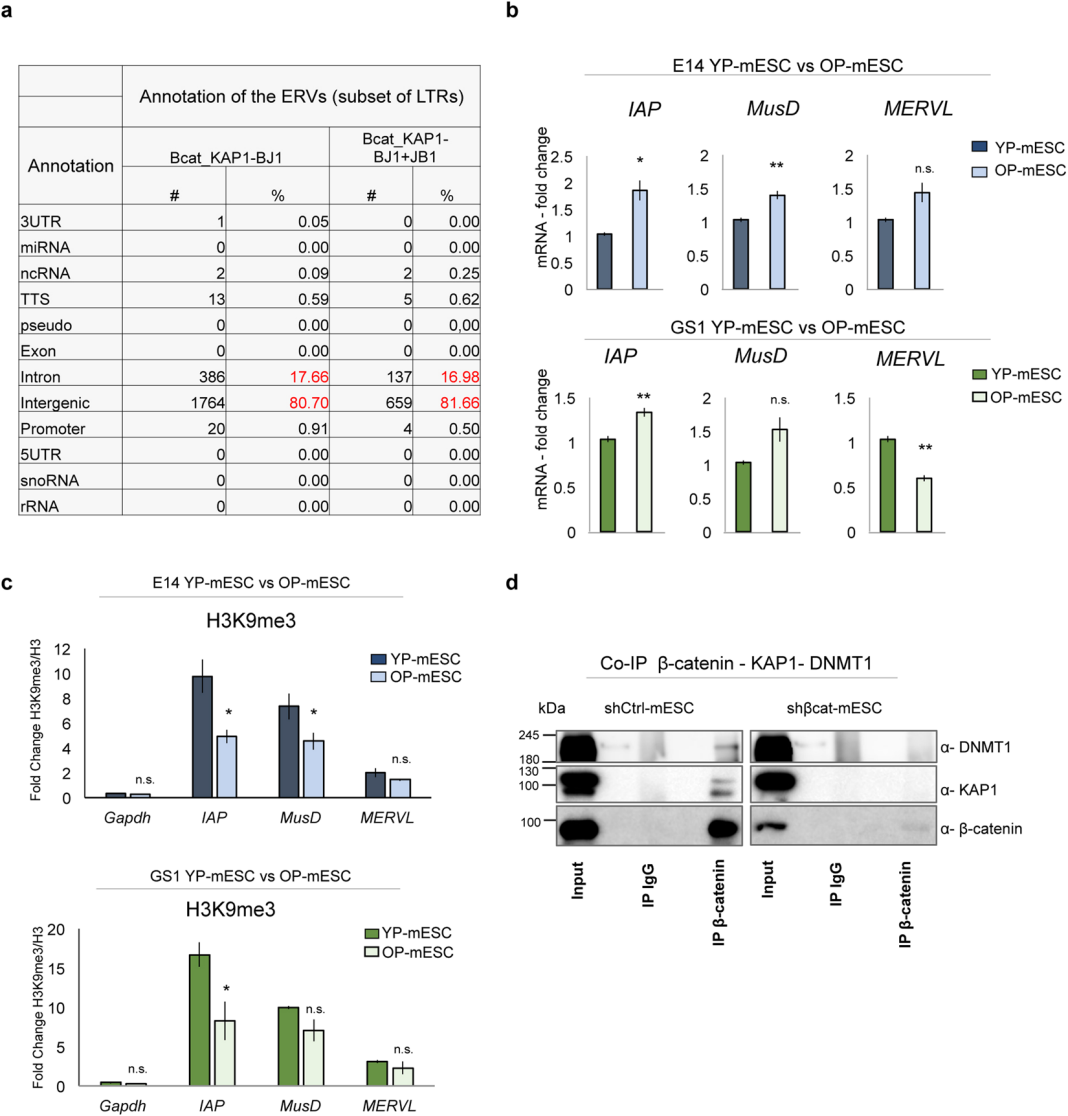
β-catenin interacts with the chromatin repressive complex. (**a**) Table showing genomic annotation of common ERVs bound by β-catenin and KAP1 in BJ1 (columns 2, 3), and among β-catenin, KAP1 in BJ1 and KAP1 in JB1 mESCs (columns 4, 5), represented as number (#) and percentage (%) over the total common peaks. (**b**) Quantitative real-time PCR experiments showing the expression profiles of ERVs (*IAP*, *MusD*, *MERVL*) in E14 (upper charts) and GS1 (lower charts) YP- and OP- mESCs. The transcriptional levels are normalized to *Gapdh* as a reference gene. Data are represented as fold change (2^−ΔΔCt^) relative to the YP-mESCs and the results are means of n = 3 independent experiments ± SE. (**c**) ChIP-qPCR analysis of H3K9me3 recruitment at *IAP*, *MusD* and *MERVL* ERVs in E14 (upper charts) and GS1 (lower charts) YP- and OP- mESCs. The data are represented as fold change (2^−ΔΔCt^) over H3 and means of n = 3 independent experiments ± SE. (**b**,**c**) Asterisks indicate statistical significance calculated by unpaired two-tailed t test analysis (n.s. not significant; *p < 0.05; **p < 0.01). (**d**) Co-immunoprecipitation of β-catenin with either KAP1 or DNMT1 followed by western-blot analysis, in shCtrl -or shβcat- transduced E14 mESCs. 10% of input was used for DNMT1, KAP1 and β-catenin IP. IgG were used as negative control. An empty well was included between each experimental condition (Input, IgG and IP) to avoid cross-contamination.

As expected most of the LTRs were found in the overlapping common regions between β-catenin and KAP1 corresponded to endogenous retroviral elements, with the ERVK family (including Intracisternal A-particle (IAP) and early transposons (ETn//MusD)) being the most represented one, followed by MaLR and ERV1 families (Fig. 4f). Accordingly, almost 80% and 17% of ERVs were located within intergenic genomic regions and introns, respectively (Fig. 5a).

### β-catenin can directly interact with KAP1 protein and regulate the expression of retrotransposons

To further investigate the epigenetic changes in YP- and OP-mESCs, we also analyzed the expression of some endogenous retroviruses, along with ICR DNA methylation. *IAP* and *MusD* subsets of ERVs were significantly upregulated in the E14 OP-mESCs (Fig. 5b, upper panel) but not *MERVL*, when compared to YP-mESCs. Interestingly, *IAP* expression increases also in GS1 OP-mESCs (Fig. 5b, lower panel), suggesting that these endogenous retroviral elements could be more active in OP- than in YP-mESCs.

Retrotransposons (such as *IAP* and *MusD*) are located within repressed genomic regions and they have been associated with induction and spreading of heterochromatin marks, such as H3K9me3^81^. Furthermore, they are controlled by KAP1-mediated repressive complexes, which prevent their activation and genomic spreading^46,79,80^. Therefore, to analyze whether the transcriptional up-regulation of the ERVs in OP-mESCs correlates with loss of heterochromatin marks, we performed ChIP- qPCR analysis for H3K9me3 in E14 and GS1 YP- and OP-mESCs. The recruitment of H3K9me3 decreased at *IAP LTR* in both E14 and GS1 OP-mESCs, and at *MusD* in E14 OP-mESCs (Fig. 5c upper and lower panels), which was consistent with their expression profile. However, the ChIP-qPCR profile did not follow *MERVL* expression changes, suggesting that other repressive mechanisms control specifically these elements in the OP-mESCs (Fig. 5b,c). In mESCs the expression of ERVs is controlled by a number of chromatin repressive factors, including KAP1, DNMT1, among others^82^. In particular, KAP1 acts as a co-repressor by facilitating the recruitment of repressive complexes at ERVs, ICRs or other silenced genomic regions^46^. Since, in our analysis almost all of the common overlapping regions corresponded to intergenic regions enriched in LTRs, we reasoned that β-catenin could interact with the chromatin repressive complex, in particular with KAP1. To confirm this hypothesis, we performed co-immunoprecipitation (CoIP) experiments in E14 YP-mESCs carrying either a short hairpin against a control sequence with no predicted genomic target (shCtrl) or against β-catenin (shβcat). In shCtrl-transduced mESCs, β-catenin was co-immunoprecipitated with KAP1 and DNMT1, suggesting that it can interact with each one of the components of the epigenetic repressive complex (Fig. 5d, left panel). As control, we performed CoIP experiments in the shβcat-infected mESCs. In absence of β-catenin neither KAP1 nor DNMT1 could be immunoprecipitated as expected (Fig. 5d, right panel). Importantly, the amount of total (as observed by the input band) and the immunoprecipitated β-catenin was much lower in the shβcat-infected mESCs, showing the high silencing efficiency (Fig. 5d, right panel).

### Inhibition of β-catenin causes impaired mESC differentiation and changes in retrotransposon expression, but it does not affect ICR methylation

**Figure 6:**
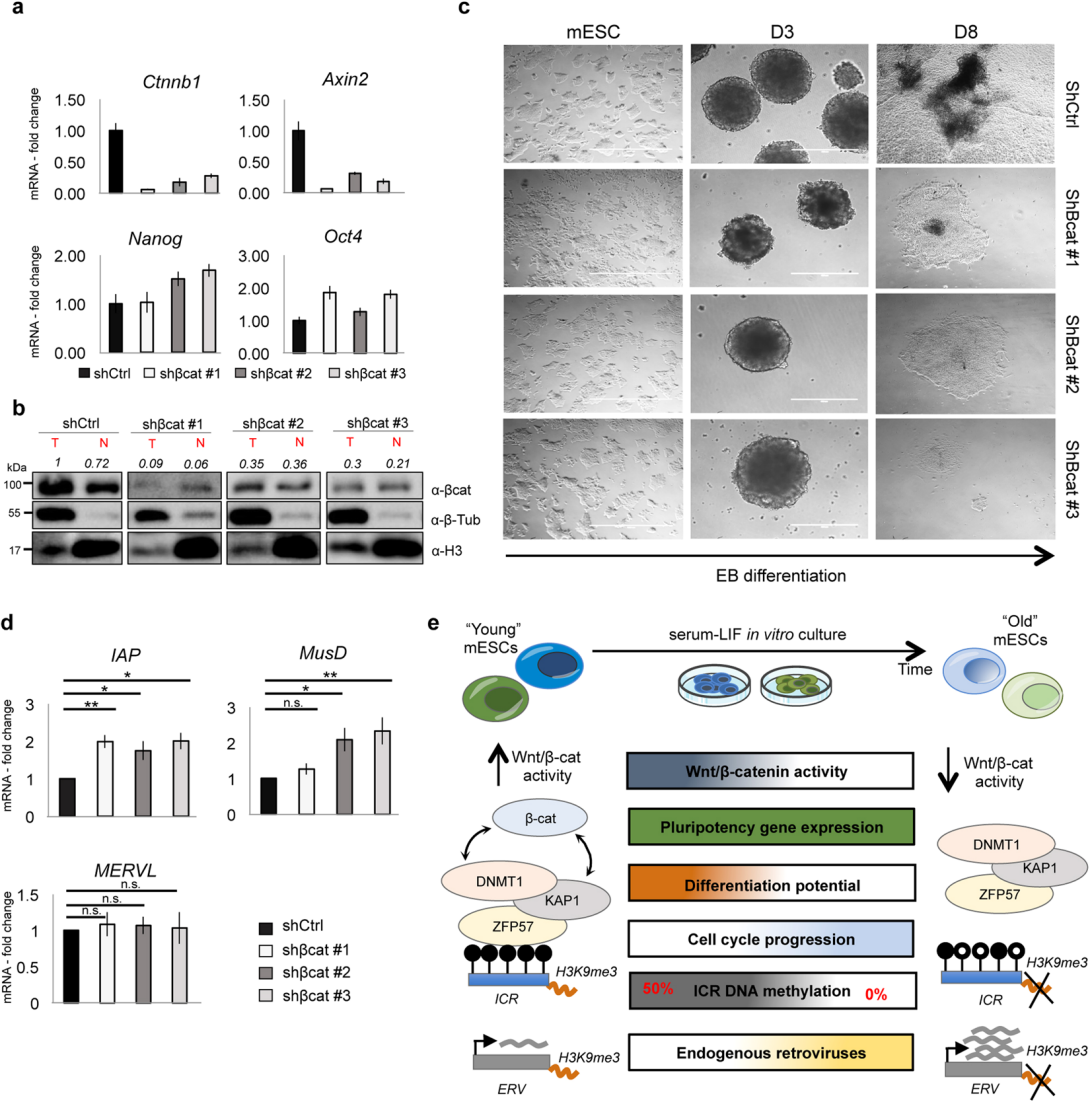
β-catenin silencing impairs mESC differentiation. (**a**) Quantitative real-time PCR analysis showing β-catenin silencing efficiency, *Axin2* and pluripotency marker (*Nanog*, *Oct4*) levels. The transcriptional levels are normalized to *Gapdh* as a reference gene. Data are represented as fold change (2^−ΔΔCt^) relative to the shCtrl-infected mESCs and the results are means of n = 3 technical replicated ± SD. (**b**) Western blot analysis showing protein levels of total and nuclear β-catenin in shCtrl-, shβcat#1-, shβcat#2- and shβcat#3- transduced E14 mESCs (n = 1). Quantification of total and nuclear β-catenin is represented as relative to total β-catenin in shCtrl-transduced mESCs. β-tubulin and H3 were used as loading controls. For western-blot quantification densitometric analysis was carried out by using ImageJ software. The quantification reflects the relative amounts as a ratio of each protein band relative to their loading control. (**c**) Representative bright field images of mESCs and embryoid bodies (EBs) at day 3 (D3), 8 (D8) after β-catenin silencing (shβcat #1, #2, #3) *versus* the control condition (shCtrl). Scale bar is 400 μm. (**d**) Quantitative real-time PCR experiments showing the expression profiles of ERVs (*IAP*, *MusD*, *MERVL*) in shCtrl-, shβcat#1-, shβcat#2- and shβcat#3- transduced E14 mESCs. The transcriptional levels are normalized to *Gapdh* as a reference gene. Data are represented as fold change (2^−ΔΔCt^) relative to shCtrl-infected mESCs and are means of n = 3 independent experiments ± SE. Asterisks indicate statistical significance calculated by unpaired two-tailed t test analysis (n.s. not significant; *p < 0.05; **p < 0.01). (**e**) Schematic representation showing cellular and epigenetic changes occurring in prolonged *in vitro* mESC cultures along with Wnt/β-catenin pathway downregulation.

To further investigate whether β-catenin plays a direct role in protecting ICRs and retrotransposons, thereby safegarding genomic stability, we knocked down β-catenin in E14 YP-mESCs by using three different pLKO-based silencing constructs^83,84^. We tested β-catenin silencing efficiency by performing qPCR and by western blot analysis. β-catenin transcript and protein (both total and nuclear) were efficiently downregulated (Fig. 6a, upper panel, and 6b), as well as the downstream target *Axin2* (Fig. 6a, upper panel). Neither the expression of pluripotency markers, *Nanog* and *Oct4* (Fig. 6a, lower panel), nor the self-renewal capacity (Fig. 6c, left micrographs) were impaired after β-catenin silencing in mESCs as expected^14,85^. Nevertheless, mESCs carrying β-catenin silencing shRNAs could not properly differentiate. The embryoid bodies started to disaggregate already at day 3 (Fig. 6c), displaying a phenotype similar with the one reported in β-catenin knockout mESCs thus confirming the already published data^14,85^.

When we analyzed DNA methylation profile by RRBS, we did not observe a drastic loss of genome-wide DNA methylation at CpG islands and ICRs, in contrast to what we observed for OP-mESCs (Fig. S5a,b, and Table S4). We performed RRBS analysis on both shCtrl- and shβcat- transduced mESCs and EBs at day 8 of differentiation. We focused on the mESCs carrying shβcat #1 construct, which showed the highest silencing efficiency. Shβcat-infected mESCs displayed lower methylation at *Peg10*, *Peg3*, *Inpp5fV2*, *Airn* (Fig. S5b, black arrows), whereas *Snrpn*, *Commd1* and *Impact* had less methylation in the shβcat-transduced EBs, when compared to control (shCtrl) (Fig. S5b, blue arrows). Moreover, we validated the RRBS data by performing COBRA analysis on several ICRs, such as *Airn*, *Grb10*, *Rasgrf1*, *Ig-DMR*, *Peg10* and *Gnas XL*. The methylation profile (as showed by the enzymatic digestion pattern) did not almost change between shCtrl- and shβcat-transduced mESCs (Fig. S5c), suggesting that the methylation differences observed in the RRBS analysis were small and not comparable to the ones observed in OP-mESCs. Nevertheless, we observed an upregulation of *IAP*, *MusD*, but not of *MERVL* expression after β-catenin silencing (Fig. 6d) suggesting that β-catenin silencing might affect the epigenetic regulation of silent genomic regions, likely acting together with repressive complexes. In conclusion, the observation that β-catenin removal did not cause relevant changes in DNA methylation might indicate that the endogenous β-catenin amount is already limiting and further reduction does not affect the methylation level.

## Discussion

Since mouse embryonic stem cells (mESCs) were isolated, in the early 80’s^6,7^, many groups started to investigate the mechanisms that define their pluripotency. Mouse ESCs present many comparable properties with the inner cell mass (ICM) of the blastocyst, such as the expression of pluripotency genes and the capacity to contribute to the formation of the three germ layers. Up to now, many studies have investigated embryonic developmental mechanisms by exploiting the capacity of mESCs to self-renew *in vitro* indefinitely. Mouse ESCs are usually cultured *in vitro* in the presence of LIF^86,87^, which directly controls the expression of the pluripotency gene-network that includes *Oct4*, *Nanog* and other factors^88^.

Nevertheless, whether the *in vitro* cultured mESCs are identical to the ICM has been a topic of debate of the last years. With the aim to obtain mESCs with features as much as possible similar to the ICM, the 2i-LIF culturing medium was designed to drive mESCs toward a naïve pluripotent state, characterized by high expression of several pluripotency genes, including *Rex1*^89,90^. This has been attributed to the activity of two small drug inhibitors (2i) of MEK1/2 and GSK3β, the latter leading to Wnt/β-catenin signaling activation. However, the identity of mESCs is not only dependent on the pluripotency gene network but also on the maintenance of a correct epigenetic state. Indeed, many groups previously observed genomic instability of mouse and human ESCs when they were cultured *in vitro* for a long time^36^. Recently, it has been shown that the constant inhibition of MEK1/2 is detrimental for mESC homeostasis after prolonged *in vitro* culture, leading to chromosomal aberrations, severe global hypomethylation and impaired differentiation capacity^39^. However, this study did not addressed whether Wnt/β-catenin activity also had an epigenetic effect in prolonged mESC cultures.

The essential role of Wnt/β-catenin signaling in mESC differentiation is widely accepted. Loss of β-catenin^12–14^ or Wnt3a^17,91^ causes embryo cell death when gastrulation starts. Likewise, we observed that OP-mESCs, which showed decreased endogenous Wnt/β-catenin activity, could not generate beating embryoid bodies with the same efficiency as YP-mESCs. On the contrary, they expressed high levels of ectodermal markers, such as *Otx2*, *Sox1*, *Pax6*, Nestin and III β-Tubulin, during both EB and neural differentiation. E14 OP-mESCs also expressed high levels of *Fgf5* during differentiation, but GS1 OP-mESCs did not, suggesting a possible epigenetic dysregulation of this gene in this strain. Since the differentiation potential of OP-mESCs was biased toward the ectodermal fate, these data might suggest that OP-mESCs could be more primed with respect to the YP-mESCs, and they could share some common features with epiblast stem cells (EpiSCs)^92,93^. It is important to note however that EpiSCs were shown as able to generate the three germ layers *in vitro* and maintain their genomic integrity^93^. In our study, the expression level of pluripotency genes was maintained high in OP-EBs, indicating that OP-mESCs could not properly exit from pluripotency state, in agreement with what was previously observed in β-catenin knockout mESCs^85^. Indeed, β-catenin knockout–derived teratomas displayed high level of pluripotency markers and features comparable to germ cell tumors.

In addition to molecular pathways alterations, several epigenetic changes have been investigated in both mouse and human ESCs. These epigenetic changes can occur both at the level of DNA methylation and histone modifications and they affect the chromatin structure and ESC identity. In particular, DNA methylation is sensitive to external stimuli and cellular stress. DNA methylation pattern is maintained stable during cell replication through the action of DNA methyltransferases (DNMTs) and other repressors, since tuned methylation levels are indispensable for mESC stability and differentiation. Loss of DNMTs leads to severe global epigenetic deregulation, which causes developmental defects and embryonic lethality, as previously reported^39,44,94–96^. Accordingly, in our study, we observed changes in DNA methylation at several CpG enriched genomic regions, which were prevalently hypomethylated upon prolonged culturing, and consequently, impaired mESC differentiation potential. Moreover, the hypomethylated CpG regions appeared to control several biological processes as indicated by the gene ontology analysis, with the metabolic processes as the most represented. Additionally, some of the analyzed hypomethylated regions were found nearby genes that have been previously reported to interact with Wnt pathway and its downstream components in different cellular contexts^97–101^. Some of these genes have been described to inhibit Wnt/β-catenin signaling pathway, such as *Grb10*, *Kdm2b*, *Tet3*, *Pcdhgc5*, *Mir148a*^97–101^. On the contrary, other hypomethylated regions corresponded to genes that act as activators or inhibitors of Wnt signaling, depending on the molecular and cellular context, such as *Runx3*, *En1*, *Nkx6-1*, *Epha2*, *Nr4a*^102–107^.

In particular, the OP-mESCs showed high hypomethylation at the level of imprinted genes, which commonly show stable allele-specific methylation pattern in the pre-implantation embryo. It is important to take into consideration that the imprinted genes are organized in clusters with a common ICR. Methylation changes of one ICR can affect the expression of many imprinted genes within the cluster (around 4–5). In this study, we observed that the YP-mESCs show 50% of methylation at many ICRs, indicating that allele specific methylation was lost in prolonged cultures, extended to both maternally and paternally imprinted loci. Additionally, the OP-mESCs were characterized by higher retrotransposon expression, such as *IAP*, when compared to YP-mESCs. Both imprinted genes and retrotransposons are tightly epigenetically controlled^27,31–34,45,46,79–81^ since they play essential roles during embryonic and extra-embryonic tissue formation. Loss of imprinting or transcriptional changes in retrotransposons, in particular in *IAP*, could indeed be detrimental to genomic stability and embryo development^26,36,39,40,94,96,108,109^. Accordingly, both E14 and GS1 OP-mESCs could not properly differentiate when compared to YP cells.

OP-mESCs were characterized by significantly faster cell cycle progression with respect to YP-mESCs, suggesting that the epigenetic changes could also affect cell cycle check-points. Interestingly, loss of methylation at the *KvDMR* ICR has been associated with lower expression of the cell cycle inhibitor cyclin-dependent kinase inhibitor 1 C, (*Cdkn1c*)^110^. In addition, Wnt/β-catenin pathway has been reported to have an anti-proliferative effect, by directly regulating the expression of cell cycle repressor genes^61^, thus safeguarding mESC identity. In line with previous published studies, we observed that both E14 and GS1 mESCs downregulated the expression of Wnt downstream targets (*Axin2*, *Lef1*, *Tcf1*, *Sp5*) and β-catenin protein at late passages. Wnt activity reduction was not associated with altered pluripotency gene expression, but it was translated into impaired differentiation potential, faster cell cycle progression and genomic instability, thus loss of mESC homeostasis. The factors causing Wnt/β-catenin pathway downregulation in prolonged cell cultures still remain unknown. Among possible triggering events, oxidative stress could be a putative inducing factor. Indeed, increased oxidative stress has been reported to antagonize Wnt signaling and has been previously described to induce genomic aberrations and loss of cell homeostasis in long-term *in vitro* cell culture^111–114^. In particular, increased oxidative stress can antagonize Wnt signaling by inducing expression of Forkhead box-O (FOXO) transcription factor^115^. FOXO competes with TCFs for its interaction with β-catenin, thus inhibiting TCF transcriptional activity and the canonical Wnt/β-catenin signaling pathway^116,117^. Additionally, among the hypomethylated regions analyzed by RRBS we found some inhibitors of Wnt/β-catenin pathway, including *Kdm2b*, which inhibits the stability of β-catenin protein^98^, thereby creating a feedback loop that could accelerate β-catenin degradation.

It is important to note that different mESC lines can display disparate endogenous levels of Wnt/β-catenin activity, due to either the diverse *in vitro* culturing conditions or different timing of mESC isolation from the embryo^20,62^. YP-GS1 mESCs showed low levels of nuclear β-catenin protein, if compared to the YP-E14 mESCs. Interestingly, ICRs, such as *Inpp5fV2* region were hypomethylated in GS1 mESCs already at early passages, suggesting that this cell line was less stable with respect to E14. The role of Wnt activity in homeostasis maintenance has been largely described in the adult stem cells. For instance, long-term hematopoietic stem cells show a decrease in the canonical Wnt/β-catenin signaling activity during ageing^118^. In parallel, other studies has reported that many epigenetic changes, including loss of imprinting, occur in hematopoietic and other adult stem cell compartments^119^. The observation that mESCs carrying gain of function Wnt mutants (S33Y-β-cat #1 and S33Y-β-cat #2) maintained normal or even higher level of methylation at the ICRs also after prolonged *in vitro* culturing, further strengthen the hypothesis of a possible “protective” role due to Wnt/β-catenin pathway, which can control epigenetic stability. Even after prolonged culturing, these clones retained Wnt/β-catenin activity and methylation at similar levels of YP-mESCs. However, it is important to take into account that additional epigenetic changes might occur in some of the clones, due to stochastic perturbations. Indeed, some of the ICRs, such as *Peg10*, *Gpr1* were hypomethylated in the OP-S33Y-β-cat #2 mESC clone, whereas others showed an increase in DNA methylation level in both β-catenin mutant clones. However, in both β-catenin overexpressing clones the overall DNA methylation profile was similar to the YP-mESCs.

Finally, we observed that β-catenin interacts with KAP1 and DNMT1 repressors, therefore it can be considered as a regulator of epigenetic stability maintenance. By analyzing published ChIP-sequencing datasets we observed that β-catenin and KAP1 share common target regions, located within intergenic regions and overlapping with endogenous retroviral elements. These data further strengthen the conclusion that β-catenin could mediate the action of repressors recruited by KAP1 on silent genomic regions, even though the potential mechanism still remains unknown. Interestingly, β-catenin protein has been previously described to interact with DNMT1 in colorectal cancer cells^120^ or with other chromatin factors in mESCs^121^. However, it is important to note that, when we silenced β-catenin in mESCs we did not detect drastic methylation changes at the ICRs, though shβcat- transduced cells upregulated the transcriptional levels of *IAP* and *MusD* endogenous retroviruses. These data suggest that either the endogenous levels of β-catenin are already limiting and its further decrease does not impair methylation maintenance, or, alternatively, that β-catenin does not directly act on the ICRs but it acts as a mediator for the repressive complexes. Up to date, the factors causing genomic instability in mESCs are not clear, even though many chromatin modifiers have been described to act on repressed genomic regions.

All in all, our data suggest that Wnt/β-catenin activity need to be maintained constantly active in mESCs during passages to ensure correct cell identity and epigenetic stability. Loss of Wnt activity results in global hypomethylated DNA, loss of chromatin repressor recruitment and activation of silent genomic regions, resulting in impaired mESC differentiation and altered cell cycle progression (Fig. 6e). Sustained activation of the Wnt signaling pathway results in maintenance of methylation at most of the ICRs after prolonged *in vitro* culture. In conclusion, Wnt/β-catenin pathway, mediates a large number of molecular and biological processes including DNA methylation at ICRs to ensure correct cell and tissue homeostasis.

## Materials and Methods

### Cell lines and differentiation protocols

GS-1 (129 Sv) and E14 (129/Ola) mouse embryonic stem cells (mESCs) were obtained from Merrill’s laboratory^122^ and purchased from ATCC, respectively. Both mESC cell lines were maintained in 0.1% gelatin (Millipore ES-006-B)-coated plates mESC medium, which consisted of DMEM supplemented with 15% fetal bovine serum (FBS), L– glutamine (2 mM), penicillin (100 U/ml), streptomycin (100 µg/ ml), sodium pyruvate (1 mM), non-essential amino acid (NEAA) (0,1 mM), 2-mercaptoethanol (0,5 mM) and ESGRO mLif (1000 U/ml). E14 mESCs were thawed at passage 8 and expanded up to passage 14 for the analysis of young passage (henceforth called YP-E14 mESCs) E14 mESCs. GS1 were thawed from passage 18 and expanded up to passage 22 for the analysis of young passage (henceforth called YP-GS1 mESCs) GS1 mESCs. To obtain the old passage (henceforth called OP-mESCs) E14 and GS1 mESCs were kept in culture for ~70 and ~50 passages, respectively, in mESC medium. At each passage cells were detached by using trypsin (0.025% trypsin and 0.04% EDTA, SIGMA 25300-054) at 37 °C, centrifuged for 5 minutes and 300 rcf and plated with a dilution ratio of 1:15–1:20 at each passage.

The differentiation medium for the production of embryoid bodies (EBs) consisted of mESC culture medium without LIF. The cells were harvested by trypsinisation, counted, and propagated in hanging drops (400 single mESCs/30 µl initial drop) for 2 days, before being transferred to 10 cm^2^ bacterial dishes, where the cells grow in suspension. On day 5, the embryoid bodies were transferred onto gelatinized p100 dishes always in differentiation medium, which consisted of mESC culture medium without the LIF. The medium was changed every 2 days and the beating embryoid bodies were observed starting from day 8 of the differentiation process. For expression profile analysis the cells were harvested and pelleted at day 0 (ESC), 6 and 12 of the differentiation process.

For neural differentiation in monolayer, undifferentiated mESCs were gently dissociated using trypsin (0.025% trypsin and 0.04% EDTA, SIGMA 25300-054) at 37 °C and plated onto 0.1% gelatin-coated tissue culture plastic at a density of 0.5–1.5 × 10^4^/cm^2^ in N2B27 medium [1:1mix of DMEM/F12 (GIBCO) supplemented with N2 (GIBCO) and Neurobasal medium (GIBCO) supplemented with B27 (GIBCO)], L–glutamine (0,5 mM), 2-mercaptoethanol (0,1 mM) and retinoic acid (1 μM). The medium was refreshed every other day^64,65^. For expression profile analysis cells were detached by using Accutase (A1110501, GIBCO) and pelleted at 300 rcf for 5 minutes.

### Total protein extraction

Cells were trypsinized (0.025% trypsin and 0.04% EDTA, SIGMA 25300-054) at 37 °C, pelleted at 300 rcf and washed twice with PBS. During each wash cells were pelleted at 300 rcf for 5 min 4 °C. Cell lysis was performed on ice for 25 min, in RIPA buffer (150 mM NaCl, 1% Nonidet P40, 0.5% sodium deoxycholate, 0.1% sodium dodecyl sulphate, 50 mM Tris-HCl, pH 8.0) containing protease (SIGMA P8340) and phosphatase inhibitors (SIGMA P2850). Insoluble material was pelleted by centrifugation at 16,000 rcf for 30 min at 4 °C. Protein concentrations were determined using the Bradford assay (Bio-Rad 500-0006). Western blot was performed as specified in the apposite section with the antibodies indicated in Table S6.

### Nuclear protein extraction

For nuclear protein extraction cells were trypsinized (0.025% trypsin and 0.04% EDTA, SIGMA 25300-054) at 37 °C, pelleted at 300 rcf and washed twice with cold PBS. During each wash cells were pelleted at 300rcf for 5 min at 4 °C. Cells were incubated in hypotonic buffer (10 mM Tris-HCl pH 7.8, 5 mM KCl, 2 mM MgCl_2_ DTT 1 mM) containing protease inhibitors (SIGMA P8340) for 10 min at 4 °C. Cells were pelleted at 300 rcf for 5 min at 4 °C and plasma membrane lysis was performed in 0,25% NP-40 hypotonic buffer on ice for 15 min. Nuclei were pelleted at 300 rcf for 15 min at 4 °C and washed twice in hypotonic buffer. Isolated nuclei were incubated in RIPA buffer (150 mM NaCl, 1% Nonidet P40, 0.5% sodium deoxycholate, 0.1% sodium dodecyl sulphate, 50 mM Tris-HCl, pH 8.0) containing protease (SIGMA P8340) and phosphatase inhibitors (SIGMA P2850). Insoluble material was pelleted by centrifugation at 16,000 rcf for 30 min at 4 °C. Protein concentrations were determined using the Bradford assay (Bio-Rad 500-0006). Western blot was performed as specified in the apposite section with the antibodies indicated in Table S6.

### Protein immunoprecipitation

For each immunoprecipitation condition 50 µl of Dynabeads (Thermo scientific 10004D) were washed 3 times in 500 µl of cold CHAPS buffer (50 mM TrisHCl pH 7.5, 150 mM NaCl, 0.15% CHAPS) containing protease inhibitors (SIGMA P8340). To separate the beads from the wash solution the tubes were placed on the magnet. The isolated beads were re-suspended in 500 µl of antibody solution containing 8 µg of antibody (DNMT1 (Abcam, ab87656) β-catenin (Millipore, 06-734) KAP1 (Abcam, ab10483),) or IgG (Abcam, ab46540) in cold CHAPS buffer containing protease inhibitors) and incubated O/N at 4 °C on a rotating wheel.

Cell fractionation and nuclei isolation was performed as described in the previous paragraph. For co-immunoprecipitation experiments, nuclei were lysed in CHAPS buffer (50 mM TrisHCl pH 7.5, 150 mM NaCl, 0.15% CHAPS containing protease inhibitors) for 15 min at 4 °C and were immerged in liquid nitrogen for 2 min and successively thawed on ice to perform a freeze-thaw lysis. Insoluble material was pelleted by centrifugation at 16,000 rcf for 30 min at 4 °C and the supernatants (100 µg of the nuclear protein extract) were incubated with antibody-coupled dynabeads overnight O/N at 4 °C. Beads were washed three times with CHAPS buffer containing protease inhibitors and elution was performed by boiling beads in Laemmli buffer (1x) at 95 °C for 10 min. Western blot was performed as specified in the apposite section with the antibodies indicated in Table S6.

### Western blot

Either total protein extract or nuclear protein extract was mixed with 4 × Laemmli buffer (40% glycerol, 240 mM Tris/HCl, pH 6.8, 8% SDS, 0.04% bromophenol blue, 5% β-mercaptoethanol) and denatured at 99 °C for 10 minutes. Either total protein extract or nuclear protein extract, or co-immunoprecipitation eluate was separated by SDS-PAGE, and transferred to poly vinylidene difluoride membrane (BIO-RAD 162-0177). The membranes were blocked with 5% non-fat dry milk (SIGMA 70166) in TBS-Tween 20 (0,1%) (SIGMA P1379) for 60 min, incubated with primary antibodies (β-catenin (BD, 610153), NANOG (Calbiochem, #SC1000), OCT-4 (Santa Cruz, sc-5279), β-tubulin (SIGMA, T0198), DNMT1 (Abcam, ab87656), KAP1 (Abcam, ab10483)) overnight at 4 °C. The working dilution of each antibody is listed in Table S6. The poly vinylidene difluoride membrane was then washed three times with TBS-T for 15 min, incubated with the peroxidase-conjugated secondary antibody (1:2000, Amersham Biosciences NA931 (Mouse IgG) and NA934 (Rabbit IgG)) in TBS-T with 5% non-fat dry milk for 60 min, and washed three times with TBS-T for 10 min. Immunoreactive proteins were detected using Pierce ECL Western Blotting Substrate (Thermo Scientific 32106). Densitometric analysis was carried-out by using ImageJ software. The quantification reflects the relative amounts as a ratio of each protein band relative to their loading control.

### Reduced representation bisulfite sequencing (RRBS) data analysis

Reads were processed by adaptor trimming (Illumina Pipeline Casava v1.8.2), filtered for low quality reads (Trim Galore v0.2.8) and subjected to quality control (FastQC). Reads were aligned using Bismark v0.7.9^124^ to the *Mus musculus* genome (assembly NCBI37/mm9). CpG methylation calls were extracted using the Bismark methylation extractor v0.7.9. The methylation level of a DNA region was defined using SeqMonk v0.32.1 pipeline (Simon Andrews, Babraham Institute, UK) considering at least 2 CpGs covered by at least 10 reads. Hypomethylated DNA regions were identified by searching for sequences with common symmetric CpGs (at least 10 CpGs covered by at least 10 reads that were less than 2 kb apart) with a decrease in methylation of >25%. Clustering and correlation analysis were performed using R package methylKit^125^. The RRBS data are available under the GSE109417 accession number.

### Analysis of published ChIP-seq data sets

The CpG islands data was downloaded from the UCSC genome annotation data-base for the July 2007 assembly of the mouse genome (mm9, NCBI build 37). The repeat types and coordinates were extracted from the RepeatMasker file (UCSC table browser). We used bedtools (v2.25.0)^126^ to overlap ChIP-seq peak data coordinates. The annotatePeaks tools from HOMER suite of programs^127^ was used to annotate the resulting peak overlaps (using mm9 version of Mus musculus genome assembly).

Relevant figures were produced in the R environment using mainly ggplot2^128^, reshape2^129^ VennDiagram^130^ and UpSetR^131^ packages.

## Supplementary information

Supplementary Information and figures

Movie S1 YP-E14

Movie S2 YP-GS1

Movie S3 OP-E14

Movie S4 OP-GS1

Table S1

Table S2

Table S3

Table S4
