# Supplementary material for: Wnt/β-catenin signaling pathway safeguards epigenetic stability and homeostasis of mouse embryonic stem cells
Source: Sci Rep. 2019 Jan 30;9:948. doi: 10.1038/s41598-018-37442-5 (PMC6353868; doi:10.1038/s41598-018-37442-5)

Inventory of Supplementary Information

Wnt/β-catenin signaling pathway safeguards epigenetic stability and homeostasis of mouse embryonic stem cells

Ilda Theka, Francesco Sottile, Marco Cammisa, Sarah Bonnin, Marta Sanchez-Delgado, Umberto di Vicino, Maria Victoria Neguembor, Karthik Arumugam Francesco Aulicino, David Monk, Andrea Riccio and Maria Pia Cosma

1. Supplementary Materials and Methods

2. Supplementary References

3. Supplementary Figure Legends

4. Supplementary Movie Legends

5. Supplementary Figures

1. Supplementary Materials and Methods

Construct preparation

Short hairpins targeting ß-catenin (shß-cat #1, #2, #3), and a short hairpin control (shCtrl) were cloned into the pLKO.1-Hygro lentiviral vector (Addgene plasmid #24150), following the manufacturer instructions (http://www.addgene.org/tools/protocols/plko/). The oligonucleotides cloned into the pLKO vector were purchased from Sigma-Aldrich. A list of the oligonucleotides used to generate the short hairpins is given in Table S5.

pCMV-dR8.9 dvpr (Addgene #8455) and pCMV-VSV-G (Addgene#8454) were used as lentiviral packaging constructs. The 7TGP lentiviral reporter was purchased from Addgene (Addgene #24305) [1]. S33Y-mutated β-catenin was cloned in the mammalian expression vector pCAG-NEO-ß-catenin-S33Y under the control of the pCAG promoter. The vector contains an IRES sequence and a Neomycin resistance cassette, to allow the selection of cells carrying the vector.

Immunofluorescence staining

For immunofluorescence staining, cells were fixed with 4% paraformaldehyde for 20 min at room temperature, and then washed twice with PBS. Fixed cells were then incubated in blocking solution containing 10% goat serum (Sigma) and 0.1% Triton X-100 (Sigma) for 1 h at room temperature. The cells were then left overnight at 4 °C in blocking solution containing the primary antibody. The primary antibodies used for immunofluorescence (β-catenin (BD, 610153), NANOG (Calbiochem, #SC1000), OCT-4 (Santa Cruz, sc-5279), Nestin (Abcam ab6142), Tuj1 (Millipore mab1637), and their working dilutions are listed in Table S6. The next day, the cells were washed three

times with PBS and then incubated with the secondary antibody for 1 h at room temperature. Goat anti-mouse IgG, goat anti-rabbit IgG, (1:1000, Life Technologies) conjugated to Alexa Fluor-488 or Alexa Fluor-594 were used as secondary antibodies. Nuclear staining was performed with DAPI (SIGMA 09542).

Virus preparation and cell infection

For mouse embryonic stem cell (mESC) infection, lentiviral particles were produced following the RNA interference Consortium (TRC) instructions for lentiviral particle production and infection in 6-well plates (http://www.broadinstitute.org/rnai/public/). Briefly 5 $\times 10^5$ HEK293T cells/well were seeded in 6-well plates in DMEM, supplied with 10% FBS, 10 u/ml penicillin, streptomycin 10µg/ml, 2 mM glutamine, 1mM sodium pyruvate and 100X non-essential amino acids. The day after plating, the cells were co-transfected with 1 µg pLKO-shCtrl, pLKO-shBcat #1, #2, #3, 750 µg pCMV-dR8.9, and 250 µg pCMV-VSV-G, using Polyfect reagent (Qiagen). The day after transfection, the HEK293T culture medium was substituted with the mESC culture medium. Then 5×10^4 mESCs/well were plated onto gelatin-coated 6-well plates the day before transduction. The lentiviral-containing medium was harvested from HEK293T cells at 48, 72 and 96 h after transfection, filtered, and added to the mESC plates. The day after transduction, these mESCs were washed twice in PBS and Hygromycin B selection (50 µg/ml) was applied. Not infected cells were used in parallel as a control to check the efficacy of Hygromycin B selection.

mESC transfection

To obtain S33Y-β-cat #1 and S33Y-β-cat #2 mESC mutant clones, $2x10^6$ mESCs cells were electroporated with 5 µg of pCAG-NEO-ß-catenin-S33Y using Amaxa reagent (Amaxa #VPD-1004) following manufacturer’s instructions and using A24 nucleofector

program. After nucleofection the cells were selected with 350 µg/ml G418 and two different mESC clones were picked S33Y-β-cat #1 (clone C12) and S33Y-β-cat #2 (clone C6) and cultured for several passages in mESCs media. The activity of Wnt/ß-catenin was measured by using the top flash (7TGP) lentiviral reporter construct that was purchased from Addgene (Addgene #24305) [1], carrying an eGFP under the control of 7xTcf promoter and puromycin resistance cassette under the SV40 promoter. Prior to the analysis of Wnt/ß-catenin activity, mESCs were selected with puromycin to ensure that all analyzed cells were carrying the reporter.

Flow cytometry analysis

For analysis of GFP+ and GFP- cells after transduction with the top flash reporter (7TGP), mESCs were detached by using trypsin (0.025% trypsin and 0.04% EDTA, (SIGMA 25300-054)), pelleted, washed once in PBS, and finally resuspended in 450µl of PBS with 5% FBS and DAPI (SIGMA 09542) solution. Not transduced cells were used as negative staining control and DAPI was used to exclude dead cells. The experiments were performed by using BD LSR Fortessa flow cytometer and analyzed with DiVa software. At least 10000 events per sample (excluding debris) were recorded for the FACS analysis. The samples were excited with the 488 nm laser. The FITC and the Per-CP-Cy5.5-A detectors were used to identify GFP+ and autofluorescence (false positive) cells, respectively. The results were graphically represented in logarithmic scale: x axis represents the Per-CP-Cy5.5-A autofluorescence signal intensity and the y axis represents the GFP signal intensity detected by the FITC detector.

E-cadherin and SSEA-1 staining was performed as previously described [4,5]. Briefly, cells were collected and incubated with Fc receptor blocking reagent (anti-Mouse CD16/CD32, eBioscience 14-0161-82) in PBS with 5% FBS solution for 10 min at 4°C.

The cells were washed once in PBS with 5% FBS. Next , the cells were incubated for 20 min at 4°C with either E-cadherin (0,5 µg/10^6 cells, Biolegend 147308) or SSEA-1 (0,5 µg/10^6 cells, Biolegend 125607) antibody in PBS with 5% FBS and DAPI (SIGMA 09542). E-cadherin or SSEA-1 expression was quantified by BD LSR Fortessa flow cytometer and analyzed by Flowjo software. Unstained cells were used as negative control and DAPI (SIGMA 09542) staining was performed to exclude dead cells from the analysis.

For cell cycle analysis cells were detached with trypsin (0.025% trypsin and 0.04% EDTA (SIGMA 25300-054)) and collected by centrifugation at 300rcf for 5 min. The cell pellet was resuspended and fixed overnight at -20°C in 3ml cold 70% ethanol. After fixation, the cells were centrifuged at 300rcf for 10 min at room temperature. The pellet was washed twice with 1ml PBS. During each wash, the cells were pelleted at 300rcf for 5 min at room temperature. Then cells were resuspended in DAPI solution (SIGMA 09542)(5µg/ml/10^6 cells in PBS) and incubated for 30 min on ice. Samples were analysed by BD LSR II and by using the commercially available Flowjo software.

RNA extraction and quantitative PCR detection of mRNA

Cells were washed with PBS, detached with trypsin (0.025% trypsin and 0.04% EDTA) and pelleted for RNA isolation. RNA was extracted and purified using RNeasy kits (Qiagen 74104), according to the manufacturer instructions. Total RNA was treated with DNAse I (Qiagen 79254) to prevent DNA contamination. The cDNA was synthetized with Oligo dT and random hexamer primers (iScript™ cDNA Synthesis Kit, BIO-RAD 1708890) starting from 1 µg mRNA, and stored at −20°C until used. Real-time quantitative PCR reactions from 8,3 ng of cDNA were set up in triplicate using a LightCycler DNA SYBR Green I Master (Roche 04707516001). The

transcriptional levels are normalized to *Gapdh* as reference gene. Data are represented as fold change ($2^{\wedge-\Delta\Delta Ct}$) and means of either technical or biological replicates ± SE. Primers were designed through the Universal ProbeLibrary Assay Design Center (Roche) and the oligos sequences with the respective Accesion Number and GenBank Entry are listed in Table S7. The oligos specific for IAP and MusD and the corresponding GenBank Entry were previously described by Guallar and colleagues [6].

Chromatin immunoprecipitation assay

For ChIP experiments against H3K9me3, H3 and ZFP57, mESCs were trypsinized and crosslinked in 1% formaldehyde for 10 min at room temperature. Crosslinking was quenched with 0.125 M glycine for 5 min. Then the pellet of cells was resuspended in lysis buffer 1 (50 mM HEPES pH 8, 10 mM NaCl, 1 mM EDTA, 10% Glycerol, 0.5% NP-40 and 0.25% Triton X-100) for 30 min at 4°C. Isolated nuclei were lysed in lysis buffer 2 (10 mM Tris–HCl pH 8.0, 200 mM NaCl, 1 mM EDTA and 0.5 mM EGTA) for 30 min at 4°C. The chromatin was sheared in sonication buffer (10 mM Tris–HCl pH 8.0, 100 mM NaCl, 1 mM EDTA, 0.5 mM EGTA, 0.1% sodium deoxycholate and 0.5% N-lauroylsarcosine) at high intensity for 2 rounds of 8 cycles each (30” ON- 30” OFF) to an average size of 100–400 bp using Bioruptor sonicator (Diagenode). The soluble material was quantified using Bradford assays.

For each IP, 100 µg of sonicated chromatin were diluted in a final volume of 500 µl with sonication buffer and with either rabbit IgG, ZFP57 (5 µg, Abcam ab4534), H3K9me3 (5 µg, Abcam 8898), H3 (5 µg Abcam ab1791) and incubated O/N at 4°C on a rotating wheel. Chromatin was precipitated with 30 µl protein A agarose beads for 2h at 4°C with rotation. The beads were then washed five times with 500 µl RIPA buffer (10 mM Tris–HCl pH 8.0, 140 mM NaCl, 1 mM EDTA, 0.5 mM EGTA, 0.1% sodium

deoxycholate, 1% Triton X-100and 0.1% SDS) and once with each of the following buffers: WASH buffer (50 mM HEPES, 0.5% sodium deoxycholate, 1% Triton X-100, 1 mM EDTA, 500 mM NaCl and 0.2% NaN3), LiCl buffer (0.25 M LiCl, 0.5% NP-40, 0.5% sodium deoxycholate, 1 mM EDTA and 10 mM Tris pH 8) and TE buffer (10 mM Tris pH 8, 1 mM EDTA). The bound chromatin was eluted in 100 µl TE buffer. Crosslinks were reversed by incubation O/N at 65°C after addition of 1 µl RNAse cocktail (Ambion) and 2 h at 50°C after addition of 2.5 µl SDS 20% + 2.5 µl 20 mg/ml proteinase K (Sigma). The DNA was extracted by using the QIAquick PCR purification Kit (Qiagen). Immunoprecipitated or 1% input DNAs were analyzed by real-time PCR using LightCycler DNA SYBR Green I Master (Roche 04707516001). Each reaction was performed in triplicate and all presented results are representative of experiments performed at least twice. Primers are listed in Table S8, which were already used by Quenneville and colleagues [36].

COBRA and pyrosequencing

Combined bisulfite and restriction analysis [2] were performed using sodium bisulfite treated genomic DNA samples and purified using EpiTect ® Bisulfite Kit (Qiagen). Bisulfite specific primers (Table S9) for each ICR were used with IMMOLASE™ Hotstart Taq polymerase (Bioline) for nested PCR (45+35 cycles) and amplicons were digested using either Taqα1 or BstUI restriction enzymes at 65 or 60 °C, respectively, for 4 hours (New England Biolabs). Differentially methylated products were resolved on 3–4% agarose gels and stained with SYBR® Safe DNA Gel Stain (ThermoFischer).

For pyrosequencing analysis, genomic DNA was bisulfite treated using EpiTect ® Bisulfite Kit (Qiagen) and used to generate PCR amplified templates in a two round PCR that were performed in the presence of 5′ biotin tagged primers (Table S9).

Pyrosequencing was carried out on PSQ HS 96 System and PyroMark MD System using Pyro Gold Reagent kits (Biotage, Uppsala, Sweden). Methylation was quantified using Pyro Q-CpG Software (Biotage, Uppsala, Sweden) that calculates the ratio of converted C's (T's) to unconverted C's at each CpG and expresses this as a percentage methylation. Average methylation across the DMR regions for all sequenced CpGs was calculated. The primers used for sequencing are listed in Table S10.

Statistical Analysis.

Averages from three independent experiments were calculated for most of the shown experiments and Student's unpaired two-tailed t-test were performed for statistical analysis. $p < 0.05$ defined statistical significance.

Table S5. List of top oligonucleotides used for cloning short-hairpins.

Short hairpin	ENSEMBL Gene_ID	Top oligo sequence (5'-3')
**sh-ßcat1 (CDS)**	ENSMUSG 00000006932	CCGG**TCTAACCTCACTTGCAATAAT**CTCG AGATTATTGCAAGTGAGGTTAGATTTTT
**sh-ßcat2 (CDS)**	ENSMUSG 00000006932	CCGG**GCTGATATTGACGGGCAGTAT**CTCG AGATACTGCCCGTCAATA
**sh-ßcat3 (3'UTR)**	ENSMUSG 00000006932	CCGG**GGCGTTATCAAACCCTAGCCTT**CTC GAGAAGGCTAGGGTTTGA
**sh-Ctrl**		CCGG**GTCACGATAAGACAATGAT**CTCGAG ATCATTGTCTTATCGTGACTTTTT

Table S5. List of top oligonucleotides used for cloning short-hairpins into pLKO-Hygro digested with AgeI/EcoRI. Sense target sequences are highlighted in bold.

Table S6. List of antibodies.

Antibodies	Working dilution/ Concentration	Company (Catalog number)
**Mouse monoclonal Antibody (Ab)**		
**E-cadherin**	0,5 µg/10^6 cells	Biolegend 147308
**SSEA-1**	0,5 µg/10^6 cells	Biolegend 125607
**NANOG**	1:300 (IF)<br>1:1000 WB	Calbiochem (#SC1000)
**OCT4**	1:200 (IF)<br>1:1000 (WB)	Santa Cruz Biotechnology (sc-5279)
**β -tubulin**	1:2000 (WB)	SIGMA (T0198)
**Tuj1**	1:200 (IF)	Millipore (mab1637)
**Nestin**	1:200 (IF)	Abcam (ab6142)
**β -catenin**	1:200 (IF)<br>1:1000 (WB)	BD (610153)
**Rabbit polyclonal Antibody (Ab)**		
**H3**	5 µg (ChIP)	Abcam (ab1791)
**H3K9me3**	5 µg (ChIP)	Abcam (ab8898)
**ZFP57**	5 µg (ChIP)	Abcam (ab45341)
**β -catenin**	8 µg (IP)	Millipore (06-734)
**IgG**	5 µg (ChIP)<br>8 µg (IP)	Abcam (ab46540)
**KAP1**	8 µg (IP)	Abcam (ab10483)
**DNMT1**	8 µg (IP)	Abcam (ab87656)

Table S6. Antibodies used in this study and the corresponding working dilution.

Table S7. List of primers used for quantitative real-time PCR.

Gene	Forward (5´→3´)	Reverse (5´→3´)	Accession Number – GenBank
Axin2	GAGAGTGAGCGG CAGAGC	CGGCTGACTCGTT CTCCT	NM_015732.4
Ctnnb1	CGACACTGCATA ATCTCCTGCTCC	GGTCCACCACTG GCCAGAATGAT	NM_001165902.1 (V1)<br>NM_007614.3 (V2)
Fgf5	AATATTTGCTGTG TCTCAGG	TAAATTTGGCACT TGCATGG	NM_010203.5
Gapdh	GTATGACTCCACT CACGGCAAA	TTCCATTCTCGGC CTTG	NM_001289726.1 (V1)<br>NM_008084.3 (V2)
Gata6	TGGCACAGGACA GTCCAAG	GGTCTCTACAGCA AGATGAATGG	NM_010258.3
Nanog	AACCAAAGGATG AAGTGCAAG	TCCAAGTTGGGTT GGTCCA	NM_028016.3 (V1)<br>NM_001289828.1 (V2)<br>NM_001289830.1 (V3)<br>NM_001289831.1 (V4)
Nkx2.5	GACGTAGCCTGG TGTCTCG	GTGTGGAATCCGT CGAAAGT	NM_008700.2
Oct4	CGTGGAGACTTTG CAGCCTG	GCTTGGCAAACT GTTCTAGCTCCT	NM_013633.3
Otx2	CATGATGTCTTAT CTAAAGCAACC	GTCGAGCTGTGCC CTAGTA	NM_001286481.1 (V1)<br>NM_001286482.1 (V2)<br>NM_144841.5 (V3)<br>NM_001286483.1 (V4)<br>NM_001360225.1 (V5)<br>NM_001360226.1 (V6)
Pax6	CCACCCATGCCCA GCTT	AACTGACACTCC AGGTGAAATGAG	NM_001244198.2 (V1)<br>NM_001244200.2 (V2)<br>NM_013627.6 (V3)<br>NM_001244201.2 (V4)<br>NM_001244202.2 (V5)<br>NM_001310144.1 (V6)<br>NM_001310145.1 (V7)<br>NM_001310146.1 (V8)
Rex1	AGGAAATAGGTA GAGCGCATCGCA	AGGCGATCCTGCT TTCTTCTGTGT	NM_009556.3

Tcf1	GCTGCCTGAGGTC AGAGAAT	CCCCAGCTTTCTC CACTCTA	NM_001313981.1 (V1) NM_009331.4 (V2)
Lef1	TGGTTAACGAGTC CGAAATCA	AGAGGACGGGGC TTGTCT	NM_010703.4 (V1) NM_001276402.1 (V2)
Sp5	ACCGGGACACTTT CGAGGCCACTCC	CAGCAGCGACTC CCACAAGCAAGG C	NM_022435.2
Sox1	GTGACATCTGCCC CCATC	GAGGCCAGTCTG GTGTCAG	NM_009233.3
IAP	TCAAGGACAGGG TATTGTTG	TCGGGTGAGTCTT TCTGGTAC	M17551
MusD	GATTGGTGGAAG TTTAGCTAGCAT	TAGCATTCTCATA AGCCAATTGCAT	BK001485
MERVL	CTCTACCCACTTG GACCATATGAC	GAGGCTCCAAAC AGCATCTCTA	Y12713

Table S7. List of primers used for quantitative real-time PCR experiments. Different splicing variants are indicated with the letter "V" followed by the corresponding number.

Table S8. List of ChIP- qPCR primers.

	FORWARD	REVERSE
Gapdh	CTCTGCTCCTCCCTGTTCCA	CCCACTCCGCGATTTTCA
Gnas	AAGGGCTTGAGAGAGGAAATGA	CCGAGACTCGCATCTTCCA
IAP LTR	CGAGGGTGGTTCTCTACTCCAT	GACGTGTCACTCCCTGATTGG
Airn	GGGCTACAAGAAAACTCAGCACTAG	GCTTTCTACCGGCCCTATCG
KvDMR	GGTCTATGATGGTGCATTTTGGT	AAGCCCACCGAAGTAATCCA
Rasfgr1	GAGCTGGGCTTACAAACTTCGT	CAGATCAAACAAGCAGGCAGAA
Inpp5fV2	AGCAGCCCTCCTCAACTGT	TCTTTCCAGGCGCTAAAGTC

Table S8. List of primers used for ChIP- qPCR gene expression analysis.

Table S9. Primers used for BS-PCR, COBRA and pyro-sequencing.

GENE	FORWARD	REVERSE
Gnas XL	GAAGAATTAGATGGGG AGGGAGG - (OUT) GTTTGTATTTTAATAGA TTAATG - (IN)	**BIO**- CTATCACCTTCCTAATTAC ACTTACCCCT – (OUT)
Ig-DMR	ATATGGATGTATTGTAA TATAGGTTAGGTGTT - (OUT) GGTATAGTTATGTGGTA TATTA - (IN)	**BIO**- AATACACCATAACATAAA CATAAAAATCCACAA - (OUT)
Airn	GGGGAATTGAGGTAAG TTAGGGTTTTT - (OUT) GAGGGGAGGATTTTGT AGATG - (IN)	**BIO**- GTTAGGGTGAAGATTTTTG GGTTATAAGA - (OUT)
Grb10	**BIO**- AAGTGTTGGGAAGTTTG TTT – (OUT)	TTCCTATTCTTACCCATCA - (OUT) TCTCTAACCATAATCACTC AA - (IN)
Peg10	GTTTTAAGTTAAGGAAA TGTTTGGAGTTGAG - (OUT) GGTGATTTGTTTTAAAG GTTTG - (IN)	**BIO**- CTACAAACAACACCCATTA CCCTAC - (OUT)
KvDMR	CCAAAACCAAAAACAT ACTCATCTTTAACC	CCTATACAAACTCACCCAA TCC
Ipp5fv2	TTAGGATTTAGAGTATT TGTAGAA – (OUT) TTTGGTAGTTTTTTGTTT ATTAAGT – (IN)	TTTACAAAAAAAATACA – (OUT) ACCCCACTAACACTTTAAC CATAAAT- (IN)

Table S9. List of primers used for nested BS-PCR, COBRA and pyro-sequencing. OUT and IN primers used for nested PCR. BIO indicates the biotinylated primer used for each region.

Table S10. List of primers used for pyrosequencing.

GENE	SEQUENCING PRIMER
Gnas XL	Fw - GGTAGAGAAAGAGTTTTAT
IG-DMR	Fw - GGTATAAGTTAAGTGTGT
Airn	Fw -AGGGTTTTATAGGAGGGAAG
Grb10	Rv - TAAATTTAATCCTAAAATTCCT
Peg10	Fw - TTTATAAGATTTAGAAATATAA
Rasfgr1	Rv - AATAAATATAAAAACAACAAC

Table S10. List of primers used for pyrosequencing. Primers used for sequencing are in the opposite strand with the respect to the biotinylated oligos.

2. Supplementary References

1 Fuerer, C. & Nusse, R. Lentiviral vectors to probe and manipulate the Wnt signaling pathway. *PLoS One* **5**, e9370, doi:10.1371/journal.pone.0009370 (2010).

2 Xiong, Z. & Laird, P. W. COBRA: a sensitive and quantitative DNA methylation assay. *Nucleic acids research* **25**, 2532-2534 (1997).

3 Ying, Q. L., Stavridis, M., Griffiths, D., Li, M. & Smith, A. Conversion of embryonic stem cells into neuroectodermal precursors in adherent monoculture. *Nat Biotechnol* **21**, 183-186, doi:10.1038/nbt780 (2003).

4 Sottile, F., Aulicino, F., Theka, I. & Cosma, M. P. Mesenchymal stem cells generate distinct functional hybrids in vitro via cell fusion or entosis. *Sci Rep* **6**, 36863, doi:10.1038/srep36863 (2016).

5 Theka, I., Sottile, F., Aulicino, F., Garcia, A. C. & Cosma, M. P. Reduced expression of Paternally Expressed Gene-3 enhances somatic cell reprogramming through mitochondrial activity perturbation. *Sci Rep* **7**, 9705, doi:10.1038/s41598-017-10016-7 (2017).

6 Guallar, D. *et al.* Expression of endogenous retroviruses is negatively regulated by the pluripotency marker Rex1/Zfp42. *Nucleic acids research* **40**, 8993-9007, doi:10.1093/nar/gks686 (2012).

7 Quenneville, S. *et al.* In embryonic stem cells, ZFP57/KAP1 recognize a methylated hexanucleotide to affect chromatin and DNA methylation of imprinting control regions. *Molecular cell* **44**, 361-372, doi:10.1016/j.molcel.2011.08.032 (2011).

3. Supplementary Figure Legends

Figure S1. Prolonged *in vitro* culture of GS1 mESCs correlates with low Wnt/β-catenin activity. (a) Schematic representation of Young (YP) and Old passage (OP) GS1 mESCs. (**b**) Representative bright field images of YP- and OP- mESCs. Round-shaped and flat colonies are indicated by white and yellow arrow, respectively. (**c**) Quantitative real-time PCR showing the expression profiles of *Axin2*, *Lef1*, *Tcf1*, *Sp5* in YP- and OP- mESCs. The transcriptional levels are normalized to *Gapdh* as reference gene. Data are represented as fold change ($2^{-\Delta\Delta Ct}$) relative to the YP- GS1 mESCs and means of n = 3 independent experiments ± SE. (**d**, **e**) Representative immunofluorescence (**d**) and confocal microphotographs (**e**) of β-catenin. Nuclear demarcation is indicated by white circle (right panel). (**f**) Western blot analysis showing total and nuclear β-catenin protein in YP- and OP- mESCs and its quantification (n = 1) relative to total β-catenin in YP-mESCs. For quantification, densitometric analysis was performed with ImageJ software. The quantification reflects the relative amounts as a ratio of each protein band relative to their loading control. (**g**) Representative immunofluorescence images showing OCT4 (green), NANOG (red) and their merge in YP- and OP- GS1 mESCs. (**h**, **i**) Representative western blot analysis (out of n = 2 independent experiments) of OCT4 and NANOG in YP- and OP- mESCs (**h**) and its quantification (**i**). Full scan blots are available in Supplementary Figure 7. Data are represented as fold change over the protein amount in YP- mESCs and means of n = 2 independent experiments ± SE. (**j-m**) FACS-plot showing the percentage of E-cadherin+ (**j**) and SSEA1+ cells (**l**) in YP- and OP- mESCs and its quantification (**k**, **m**) as means of 3 technical replicates ± SE (NS: non stained). (**n**,

o) Representative cell cycle FACS profile analyzed with Flowjo software (**n**) in YP- and OP- mESCs and its quantification (**o**) represented as percentage of total cells and means of n = 3 independent experiments ± SE. Scale bar is 400 μm (**b**), 200 μm (**d**, **g**) and 10 μm (**e**). (**f**, **h**) β-tubulin and H3 were used as loading controls. (**d**, **e** left panel**,** and **g**) Nuclei were stained with DAPI. (**c**, **k**, **m**, **o**) Asterisks indicate statistical significance calculated by unpaired two-tailed t test analysis (n.s. not significant; *p-value< 0.05; **p < 0.01; *** p-value < 0.001).

Figure S2. Old GS1 mESCs show differentiation defects and loss of methylation at *KvDMR* and *Inpp5fV2* ICRs. (a) Schematic representation of embryoid body (EB) differentiation protocol of YP and OP GS1 mESCs. **(b)** Representative bright field images of EBs at day 4 and 9 obtained from either YP- or OP- GS1 mESCs. Scale bar is 400 μm. (**c**) Quantitative real-time PCR showing the expression profiles of differentiation genes (*Nkx2.5*, *Gata6*, *Otx2*) and pluripotency genes (*Rex1*, *Oct4*, *Nanog*) in YP- and OP- GS1 mESCs (ESC) and during EB differentiation at day 6 (D6) and day 12 (D12). **(d)** Schematic representation showing neural differentiation protocol of Young and Old GS1 mESCs. (**e**) Quantitative real-time PCR experiment showing the expression profiles of *Sox1* at day 3 (D3) of N2B27+ retinoic acid (RA) treatment in YP- and OP- GS1 mESCs (ESC). (**f**) Representative immunofluorescence images showing Nestin (left panels) and III β-tubulin (TUJ1, right panels) protein expression in YP- and OP-mESCs at day 8 (D8) of neural differentiation. (**g**) Quantitative real-time PCR experiment showing the expression profiles of *Pax6* and *Fgf5* at day 8 (D8) of N2B27+ retinoic acid (RA) treatment in YP- and OP- GS1 mESCs. **(c, e**, **g**) The transcriptional levels are normalized to *Gapdh* as a reference gene. Data are represented as fold change ($2^{\wedge -\Delta\Delta Ct}$) relative to the YP-GS1 mESCs and the results are means of

n = 3 independent experiments ± SE (**c**, **e**) and means of n = 3 technical replicated for SD (**g**). (**c, e**) Asterisks indicate statistical significance calculated by unpaired two-tailed t test analysis (n.s. not significant; *p < 0.05; **p < 0.01; ***p-value < 0.001). (**h**) Methylation profile in YP and OP E14 mESCs analyzed by COBRA. The product of Nested-PCR on bisulfite converted genomic DNA was digested either with Taq1 or BstU1 restriction enzymes to distinguish un-methylated (U) and methylated (Me) DNA. Mouse genomic DNA from wild type mice tail and ZFP57 KO mESCs were used as positive and negative controls, respectively. Full electrophoresis scans are available in Supplementary Figure 8 (**i**) Pyrosequencing analysis confirming COBRA profile performed in E14 mESCs. Data are represented as means of methylation levels of at least 3 sequential CpG/ICR ± SE. (**j**, **k**) Bisulfite PCR sequencing of *KvDMR* and *Inppf5V2* ICRs in YP and OP E14 (**j**) and GS1 (**k**) mESCs. The CpG dinucleotides are indicated by the grey arrow and the C converted into T are highlighted in pink.

Figure S3. Old S33Y #1 and #2 mESC clones show 50% of methylation at several ICRs. (**a**) Representative FACS-plot showing the percentage of positive mESCs for 7TGP topflash reporter activity in E14-mESCs treated with either DMSO or CHIR (3 μM) for 24h. The non infected (NI) cells were used as negative control (Ctrl-). The FITC and the Per-CP-Cy5.5-A detectors were used to identify GFP+ (y axis) and autofluorescence (false positive) cells (x axis). The number of recorded events is 20000 for all conditions. Data are represented as means of n = 3 technical replicates ± SD. (**b**) PCA methylation analysis for YP-mESCs, OP-mESCs, OP-S33Y #1 and OP-S33Y #2 mESC clones. (**c**) CpG base Pearson correlation coefficient between biological replicates of the same cell line and among different cell lines. (**d, e**) DNA methylation profile (un-methylated (U) and methylated (Me)) in OP-S33Y#1 and OP-S33Y #2

mESC clones analyzed by COBRA (**d**) and pyrosequencing (**e**). Full electrophoresis scans are available in Supplementary Figure 8. The product of Nested-PCR on bisulfite converted genomic DNA was digested either with Taq1 or BstU1 restriction enzymes. (**f**) Representative bright field images showing cell morphology of OP-S33Y #1 and OP-S33Y #2 mESC. Scale bar is 200 µm.

Figure S4. β-catenin and KAP1 overlapping binding sites are located within intergenic regions and LTRs. (**a, b**) Representative ChIP-qPCR experiment (out of n=2 independent experiments) of ZFP57 (**a**) and H3K9me3 (**b**) recruitment at several ICRs in GS1 mESCs. The data are represented as fold change ($2^{\wedge-\Delta\Delta Ct}$) over IgG (**a**) or H3 (**b**) and means ± SD. (**c**, **d**, **e**) Bar chart showing the number of common regions between KAP1-β-catenin, which are bound also by H3K9me3 (**c**), H3K4me3 (**d**), or located in CpG islands (**e**). The number of common overlapping peaks is indicated on top of the bars. (**f**) Tables showing the different types of repeats represented as number and percentage (%) over Repeat masker (columns 2, 3) and over the total number of common overlapping peaks among β-catenin, KAP1 in BJ1 and KAP1 in JB1 mESCs (columns 4, 5). (**g**) Table showing genomic annotation of common LTRs bound by β-catenin and KAP1 in BJ1 (columns 2, 3), and among β-catenin, KAP1 in BJ1+ KAP1 in JB1 mESCs (columns 4, 5) represented as number (#) and percentage (%) over the total common peaks.

Figure S5. β-catenin silencing induces *IAP* transcription but does not strongly affect the ICR methylation. (**a**) Box-plot, from min–max values, showing the distribution of mCpG levels at ICRs in shCtrl- or shβcat#1- transduced mESCs and embryoid bodies (EBs) determined by RRBS analysis. The plots indicate the first

quartile, median (black line) and third quartile. Data are obtained from the average of n = 2 biological replicates. (**b**) Heat-map representation of ICR methylation levels in shCtrl- or shβcat#1- transduced mESCs or EBs. ICRs that show reduction on methylation in mESCs and EBs are indicated in black and blue arrow, respectively. (**c**) Methylation profile in either shCtrl- or shβcat#1- transduced E14 mESCs analyzed by COBRA. The product of Nested-PCR on bisulfite converted genomic DNA was digested either with Taq1 or BstU1 restriction enzymes to distinguish un-methylated (U) and methylated (Me) DNA. Full electrophoresis scans are available in Supplementary Figure 8.

Figure S6. Full scan blots relative to main figures indicated. Figures 1f, 1h, 5d, 6b were obtained by using Amersham Imager 600. Molecular weights are indicated in kDa. Boxes show cropped images used in figures.

Figure S7. Full scan blots relative to supplementary figures indicated. Figure S1f, is obtained by using Amersham Imager 600. Molecular weights are indicated in kDa. Boxes show cropped images used in figures.

Figure S8. Full electrophoresis scans relative to supplementary figures indicated. 3% agarose gel, referring to COBRA analysis shown in figures S2h, S3d and S5c. 100 bp marker was used. Boxes show cropped images used in figures.

4. Supplementary Movies legends.

Movie S1. **Time lapse analysis of embryoid bodies (EBs) derived from young passage (YP) E14 mESCs**. The movie shows one beating embryoid body at day 12 of differentiation. Scale bar is 1000 μm. Each frame has been taken every 10 seconds. Time is shown as minutes:seconds (mm:ss).

Movie S2. Time lapse analysis of embryoid bodies (EBs) derived from young passage (YP) GS1 mESCs. The movie shows one beating embryoid body at day 12 of differentiation. Scale bar is 1000 μm. Each frame has been taken every 10 seconds. Time is shown as minutes:seconds (mm:ss).

Movie S3. Time lapse analysis of embryoid bodies (EBs) derived from old passage (OP) E14 mESCs. The movie shows embryoid bodies at day 12 of differentiation: no beating EBs derived from OP-mESCs were observed at this stage. Scale bar is 1000 μm. Each frame has been taken every 10 seconds. Time is shown as minutes:seconds (mm:ss).

Movie S4. Time lapse analysis of embryoid bodies (EBs) derived from old passage (OP) GS1 mESCs. The movie shows embryoid bodies at day 12 of differentiation: no beating EBs derived from OP-mESCs were observed at this stage. Scale bar is 1000 μm. Each frame has been taken every 10 seconds. Time is shown as minutes:seconds (mm:ss).

5. Supplementary Figures

Figure S1

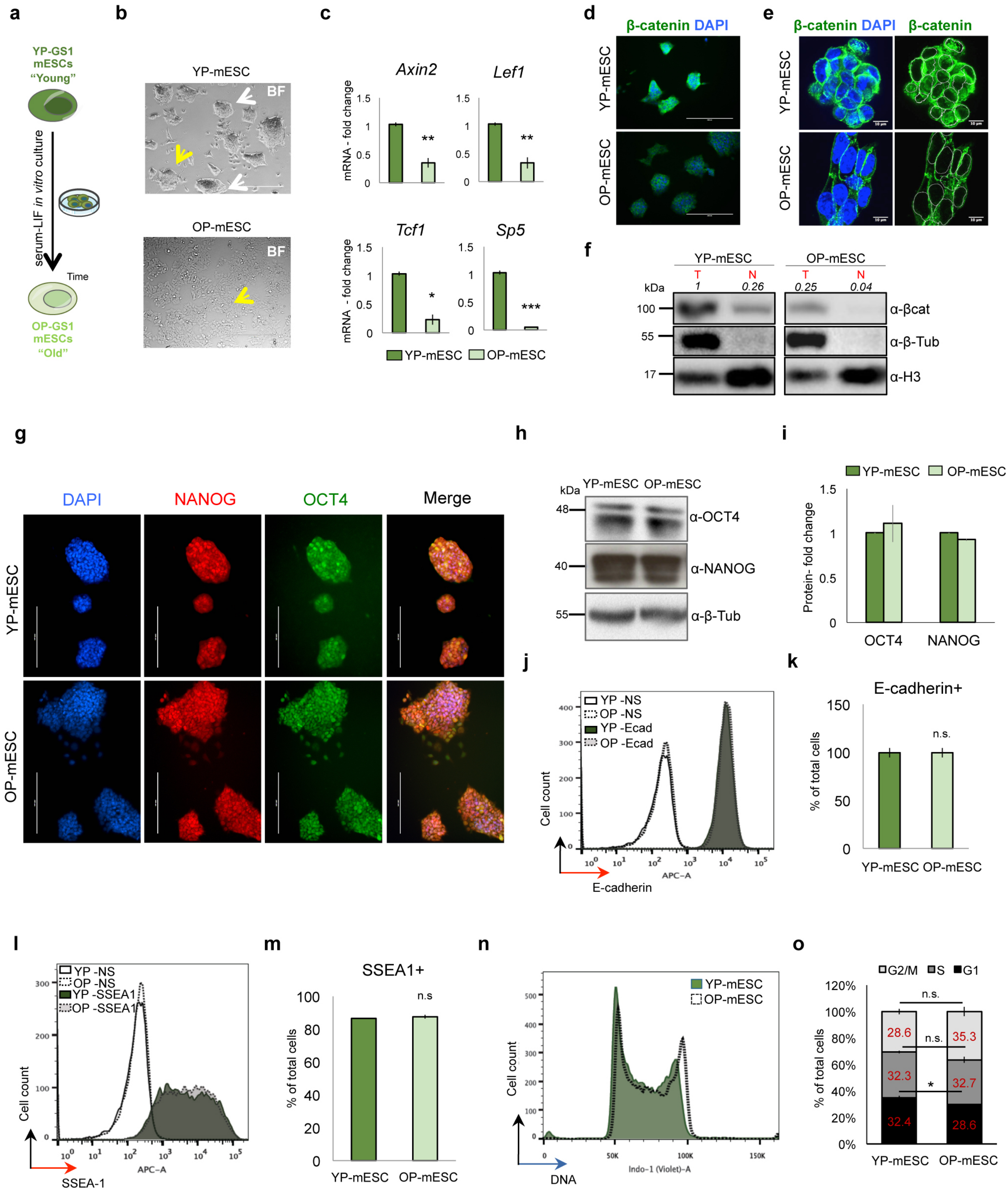

Figure S2

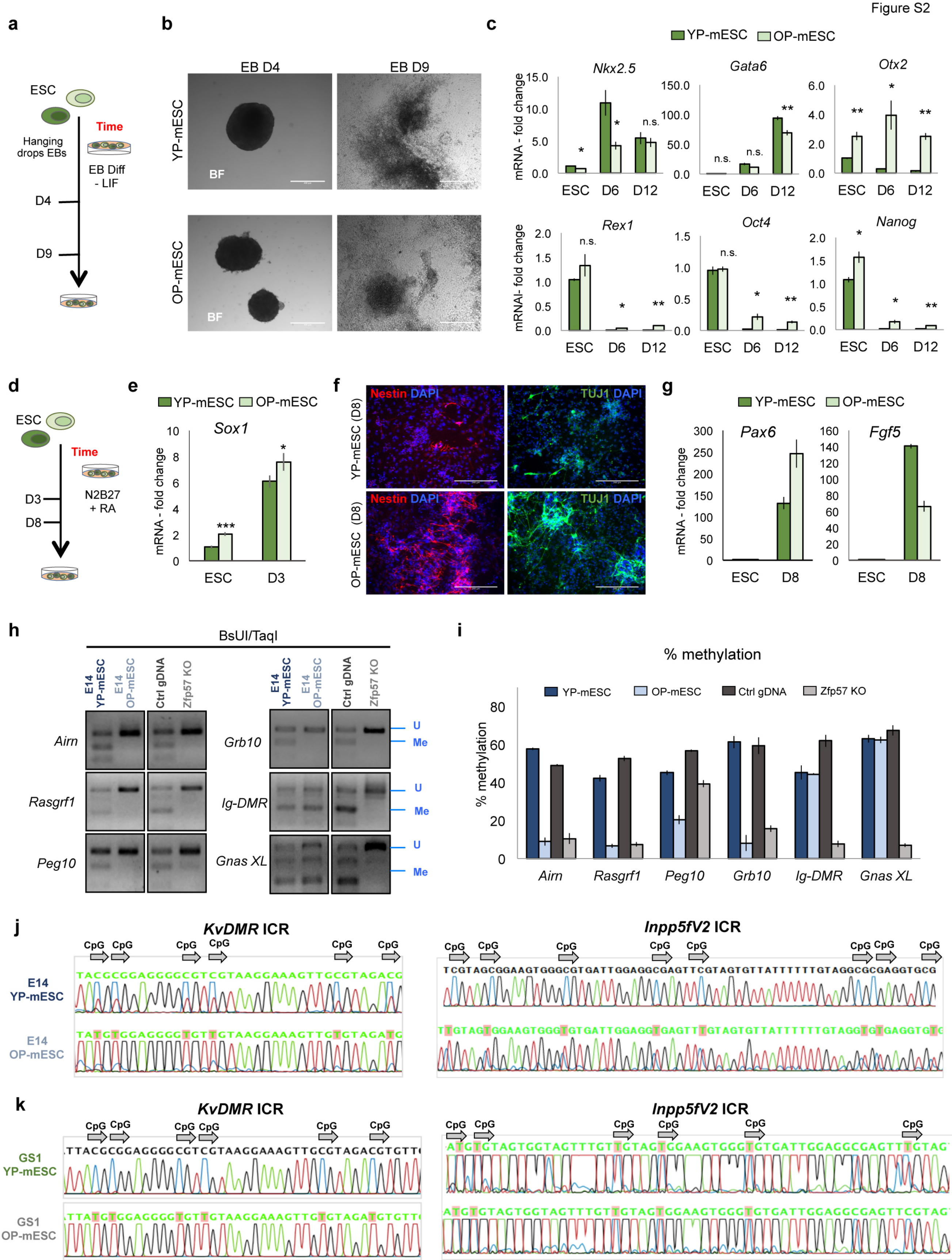

a
ESC
Time
Hanging drops EBs
EB Diff - LIF
D4
D9
b
EB D4
EB D9
YP-mESC
OP-mESC
BF
c
YP-mESC
OP-mESC
Nkx2.5
Gata6
Otx2
Rex1
Oct4
Nanog
mRNA - fold change
mRNAl- fold change
n.s.
ESC
D6
D12
d
ESC
Time
N2B27 + RA
D3
D8
e
YP-mESC
OP-mESC
Sox1
mRNA - fold change
ESC
D3
f
Nestin DAPI
TUJ1 DAPI
YP-mESC (D8)
OP-mESC (D8)
g
YP-mESC
OP-mESC
Pax6
Fgf5
mRNA - fold change
ESC
D8
h
BsUI/TaqI
E14 YP-mESC
E14 OP-mESC
Ctrl gDNA
Zfp57 KO
Airn
Rasgrf1
Peg10
Grb10
Ig-DMR
Gnas XL
U
Me
i
% methylation
YP-mESC
OP-mESC
Ctrl gDNA
Zfp57 KO
% methylation
Airn
Rasgrf1
Peg10
Grb10
Ig-DMR
Gnas XL
j
KvDMR ICR
Inpp5fV2 ICR
CpG
E14 YP-mESC
E14 OP-mESC
k
KvDMR ICR
Inpp5fV2 ICR
CpG
GS1 YP-mESC
GS1 OP-mESC

Figure S3

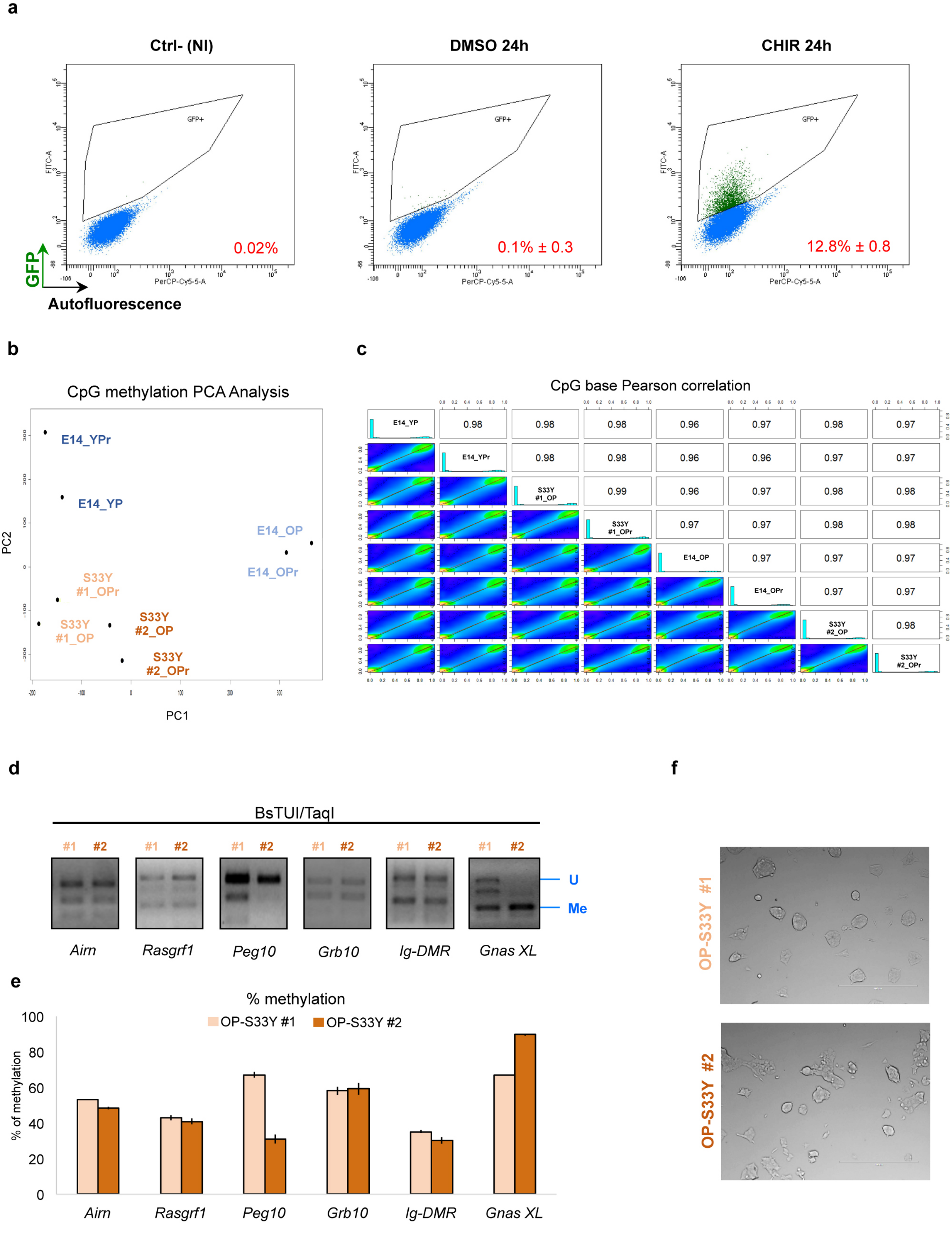
a
Ctrl- (NI)
DMSO 24h
CHIR 24h
GFP+
FITC-A
PerCP-Cy5-5-A
0.02%
0.1% ± 0.3
12.8% ± 0.8
GFP
Autofluorescence
b
CpG methylation PCA Analysis
E14_YPr
E14_YP
E14_OP
E14_OPr
S33Y #1_OPr
S33Y #1_OP
S33Y #2_OP
S33Y #2_OPr
PC1
PC2
c
CpG base Pearson correlation
E14_YP
E14_YPr
S33Y #1_OP
S33Y #1_OPr
E14_OP
E14_OPr
S33Y #2_OP
S33Y #2_OPr
d
BsTUI/TaqI
#1
#2
U
Me
Airn
Rasgrf1
Peg10
Grb10
Ig-DMR
Gnas XL
e
% methylation
OP-S33Y #1
OP-S33Y #2
% of methylation
100
80
60
40
20
0
Airn
Rasgrf1
Peg10
Grb10
Ig-DMR
Gnas XL
f
OP-S33Y #1
OP-S33Y #2

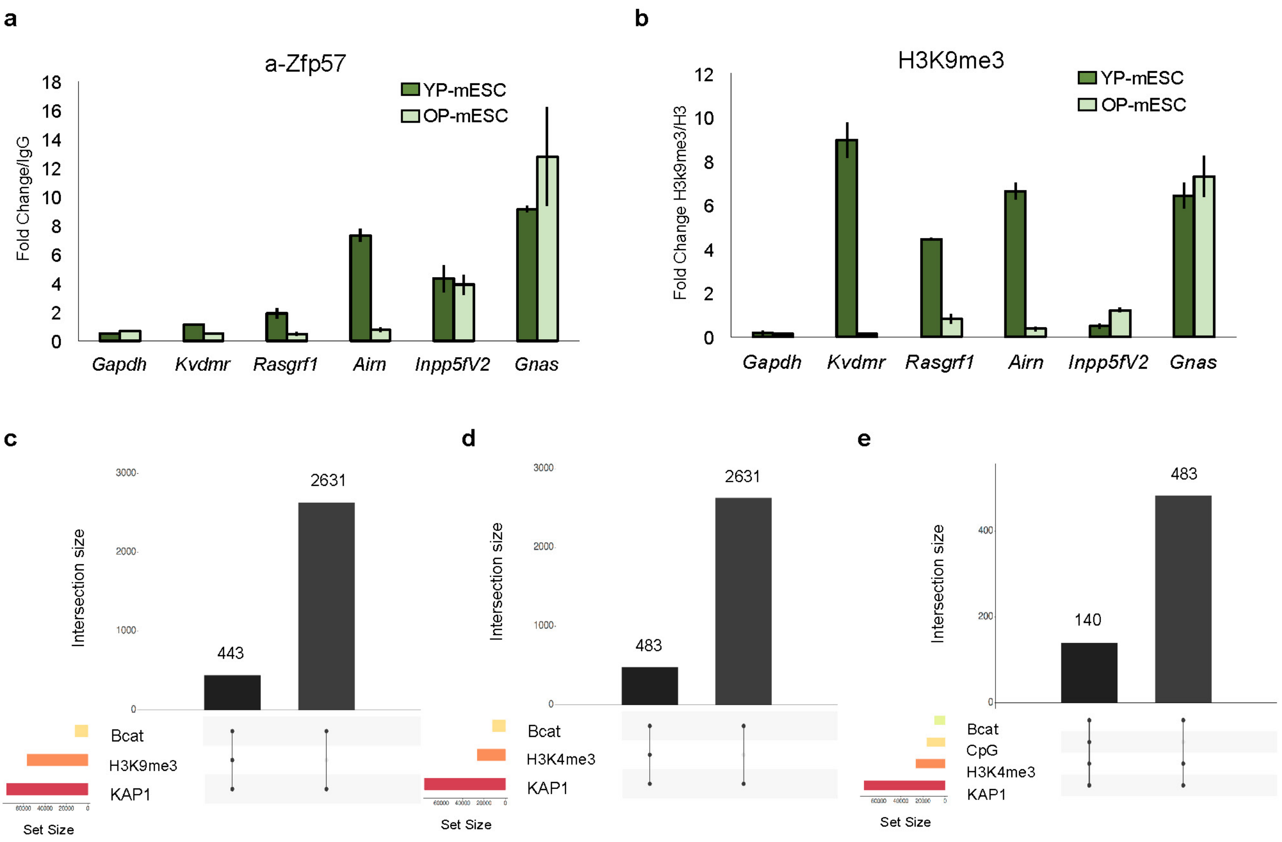

f

overlap Bcat KAP1 BJ1 / KAP1 JB1				
repeat type	all repeat masker file	% to total (all repeats)	overlap Bcat_KAP1 / repeat masker	% to total (Bcat_KAP1/repeat)
DNA	145523	3.21	46	2
LINE	758410	16.73	303	13.2
Low complexity	369358	8.15	89	3.88
LTR	692700	15.28	934	40.7
Other	159697	3.52	4	0.17
RC	404	0.01	0	0
RNA	670	0.01	1	0.04
rRNA	1548	0.03	1	0.04
satellite	7533	0.17	0	0
scRNA	8138	0.18	2	0.09
Simple repeat	984388	21.71	384	16.73
SINE	1390107	30.66	524	22.83
snRNA	2996	0.07	1	0.04
srpRNA	278	0.01	1	0.04
tRNA	5145	0.11	2	0.09
Unknown	7353	0.16	3	0.13

g

	Annotation of all LTRs			
Annotation	Bcat_KAP1-BJ1		Bcat_KAP1-BJ1-JB1	
	#	%	#	%
3UTR	7	0.25	0	0.00
miRNA	2	0.07	0	0.00
ncRNA	6	0.21	2	0.21
TTS	19	0.68	7	0.75
pseudo	0	0.00	0	0.00
Exon	0	0.00	0	0.00
Intron	545	19.42	174	18.63
Intergenic	2200	78.38	745	79.76
Promoter	28	1.00	6	0.64
5UTR	0	0.00	0	0.00
snoRNA	0	0.00	0	0.00
rRNA	0	0.00	0	0.00

Figure S5

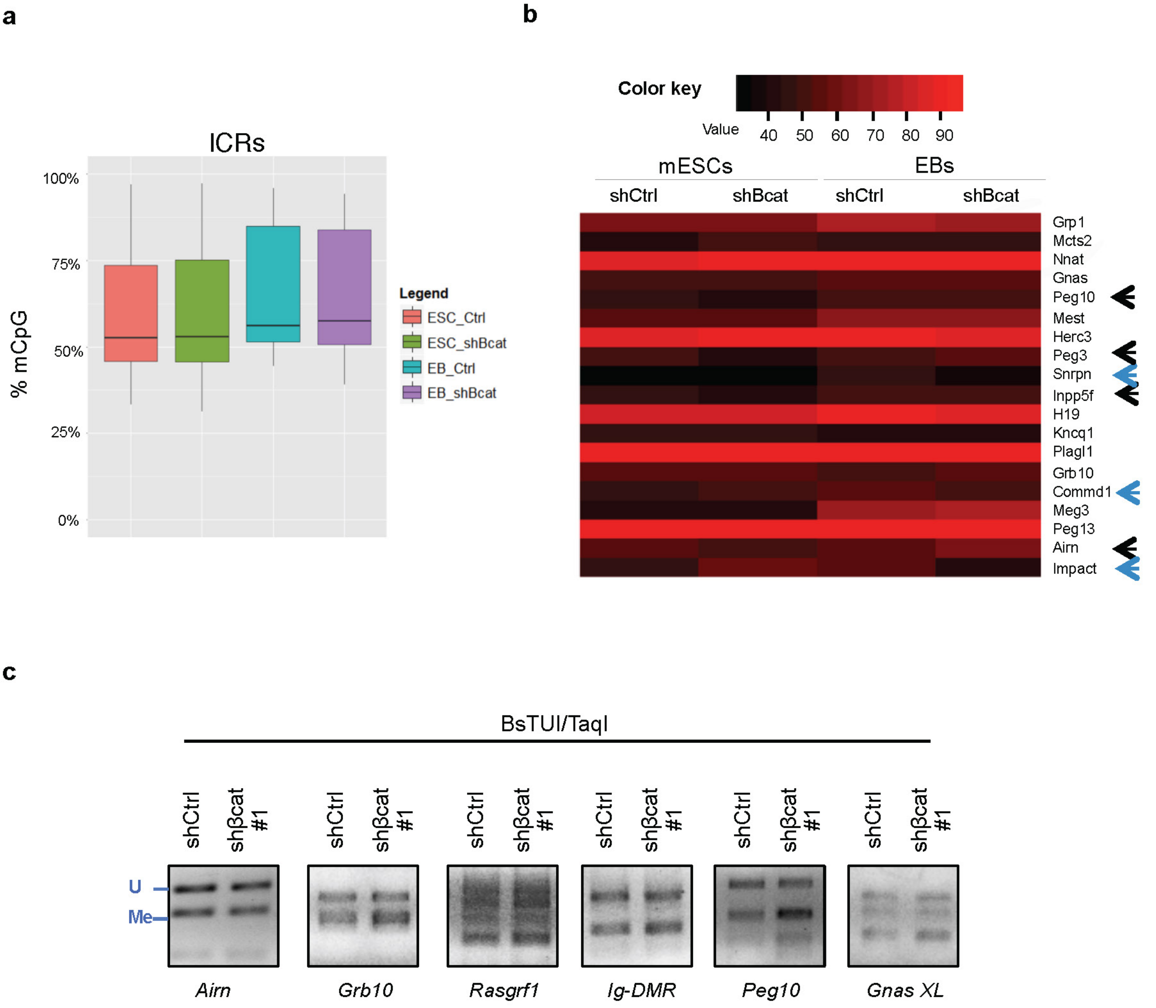

a
ICRs
100%
75%
50%
25%
0%
% mCpG
Legend
ESC_Ctrl
ESC_shBcat
EB_Ctrl
EB_shBcat
b
Color key
Value
40
50
60
70
80
90
mESCs
EBs
shCtrl
shBcat
shCtrl
shBcat
Grp1
Mcts2
Nnat
Gnas
Peg10
Mest
Herc3
Peg3
Snrpn
Inpp5f
H19
Kncq1
Plagl1
Grb10
Commd1
Meg3
Peg13
Airn
Impact
c
BsTUI/TaqI
shCtrl
shβcat #1
U
Me
Airn
Grb10
Rasgrf1
Ig-DMR
Peg10
Gnas XL

Fig. S6

Referring to Fig. 1f

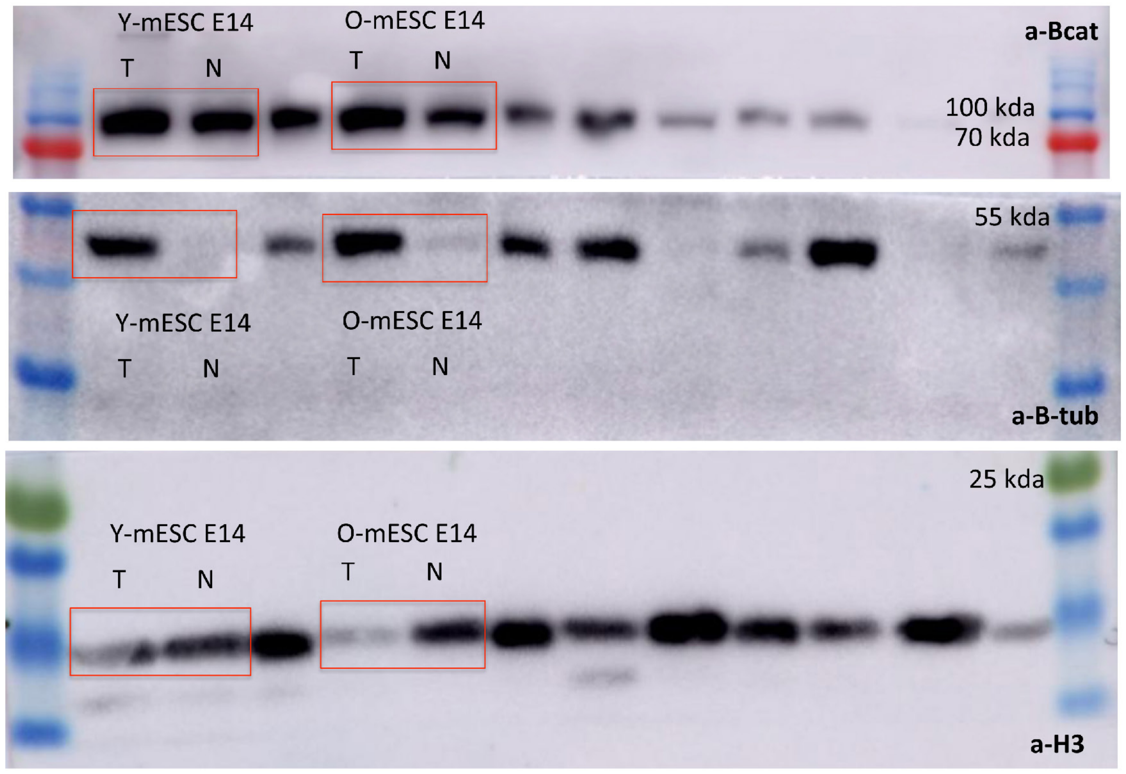

Referring to Fig. 1h

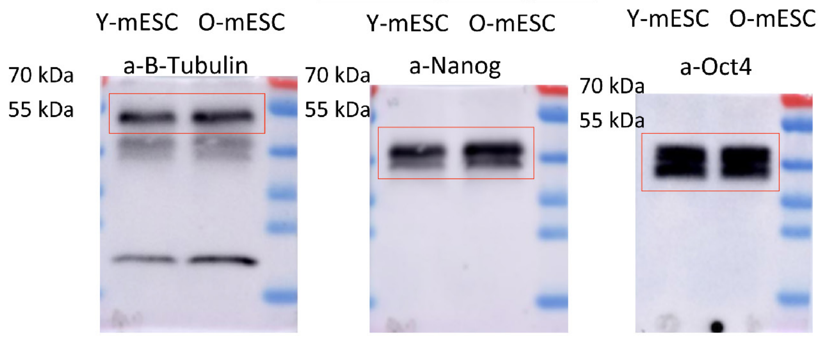

Referring to Fig. 3d

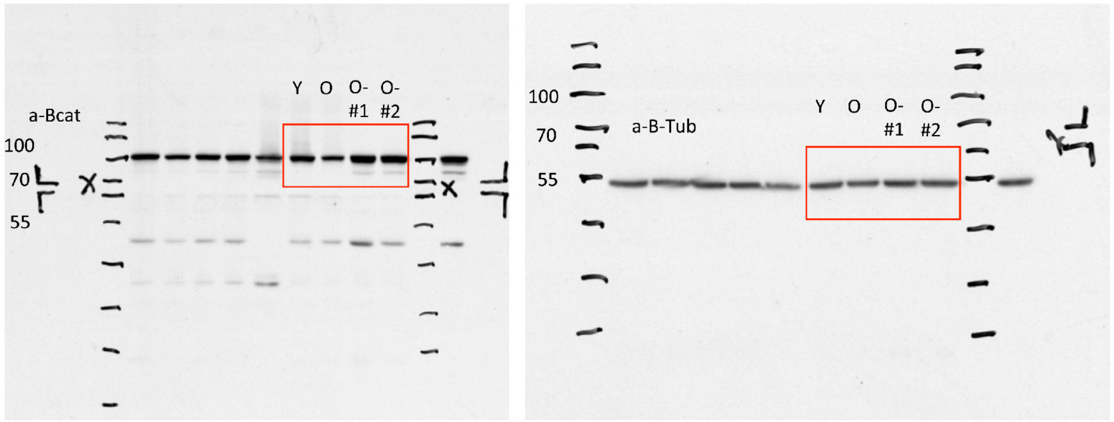

Referring to Fig. 5d

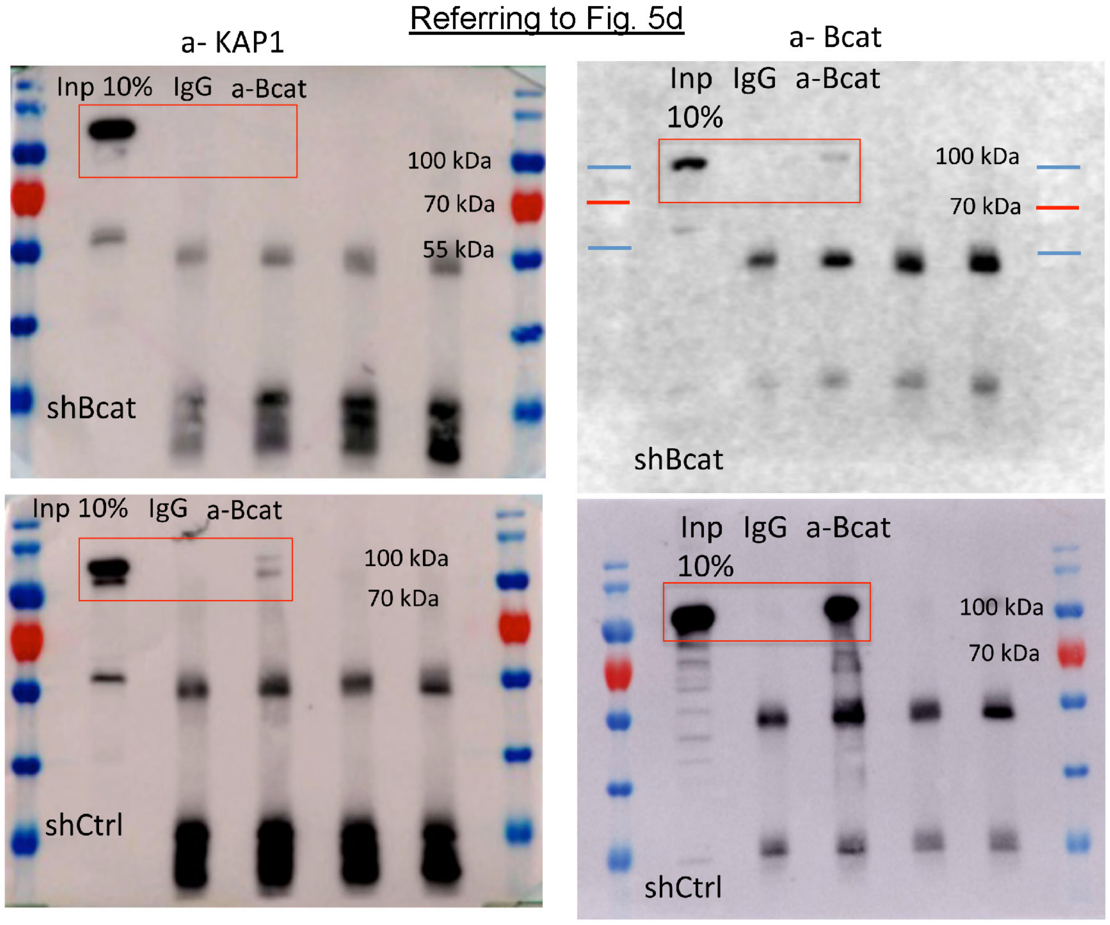

Referring to Fig. 5d

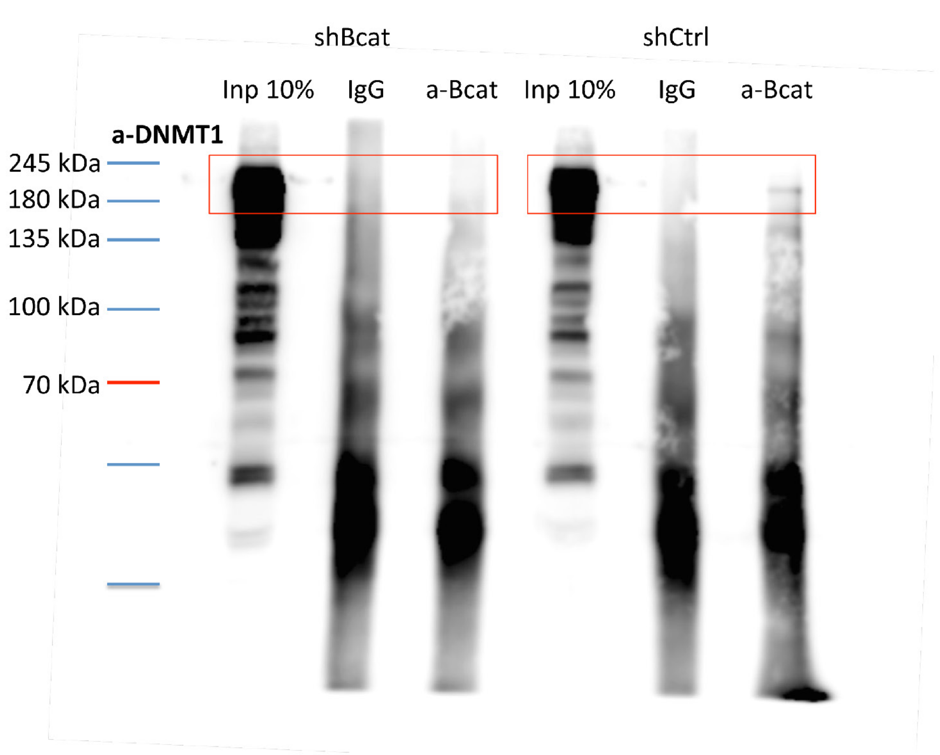

Referring to Fig. 6b

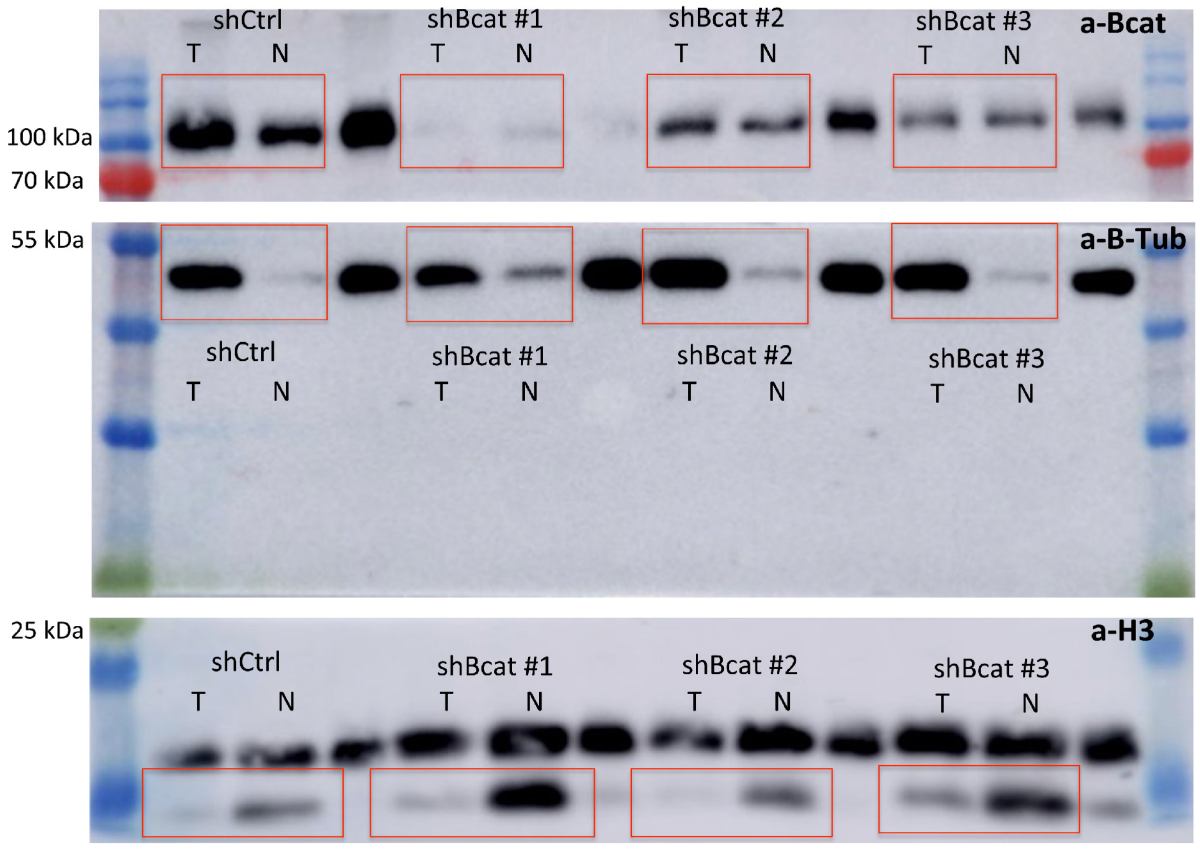

Referring to Fig. S1f

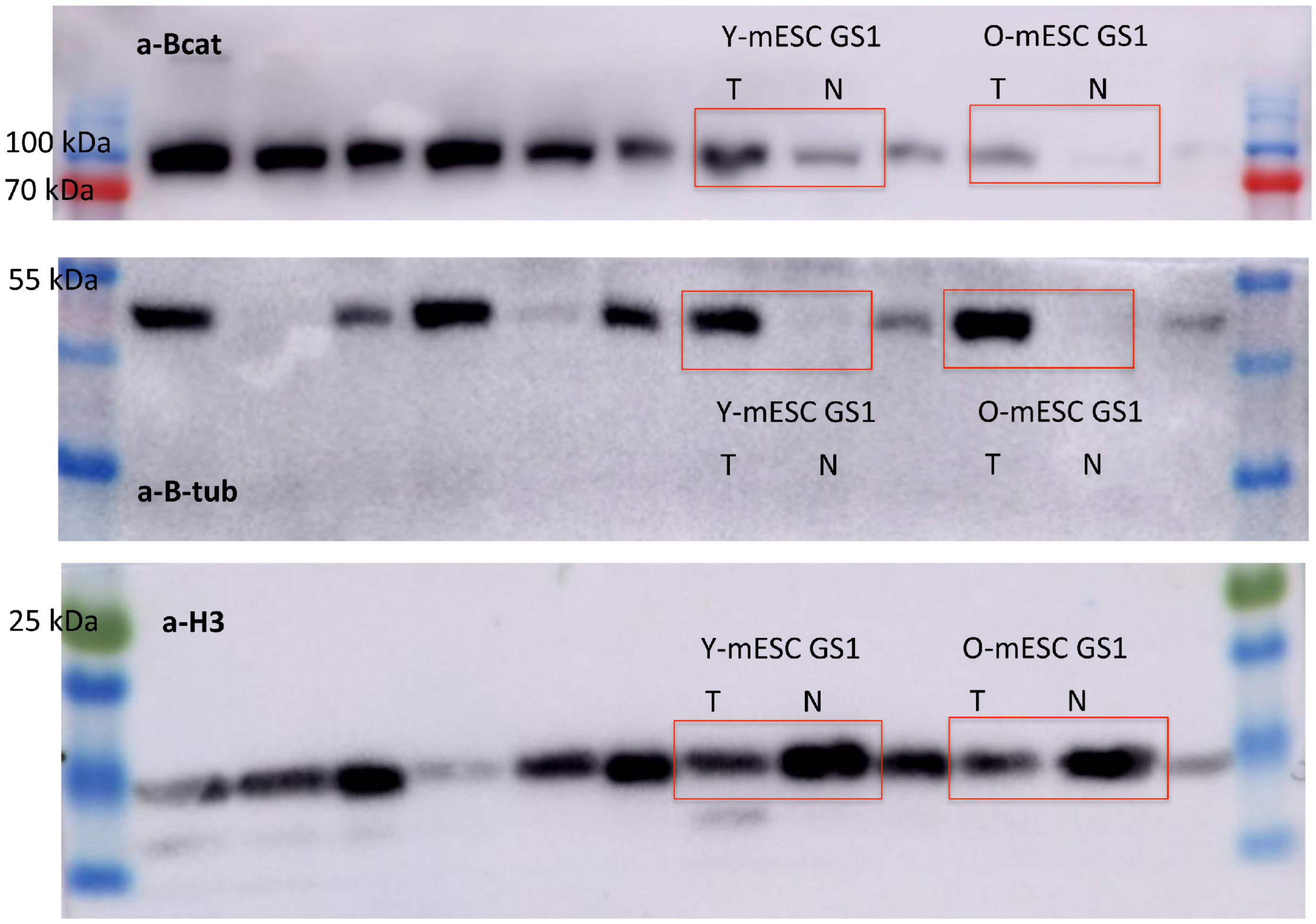

Referring to Fig. S1h

GS1-mESC

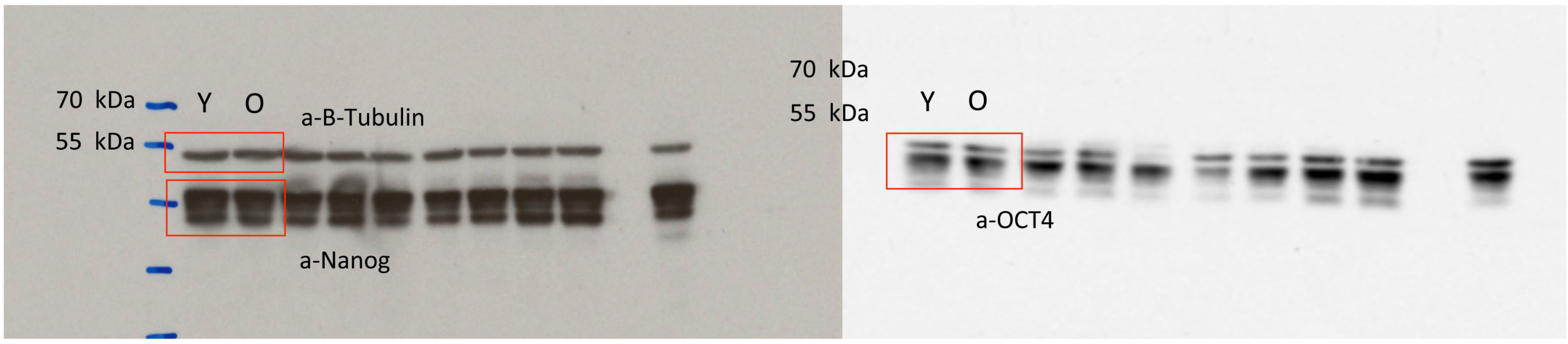

Referring to Fig. S2h and S3d

Taq1 Digestion

1- E14 Y-mESC
2- E14 O-mESC
3- S33Y #1_O
4- S33Y #2_O
5 – Ctrl gDNA
6 –- Zfp57 KO

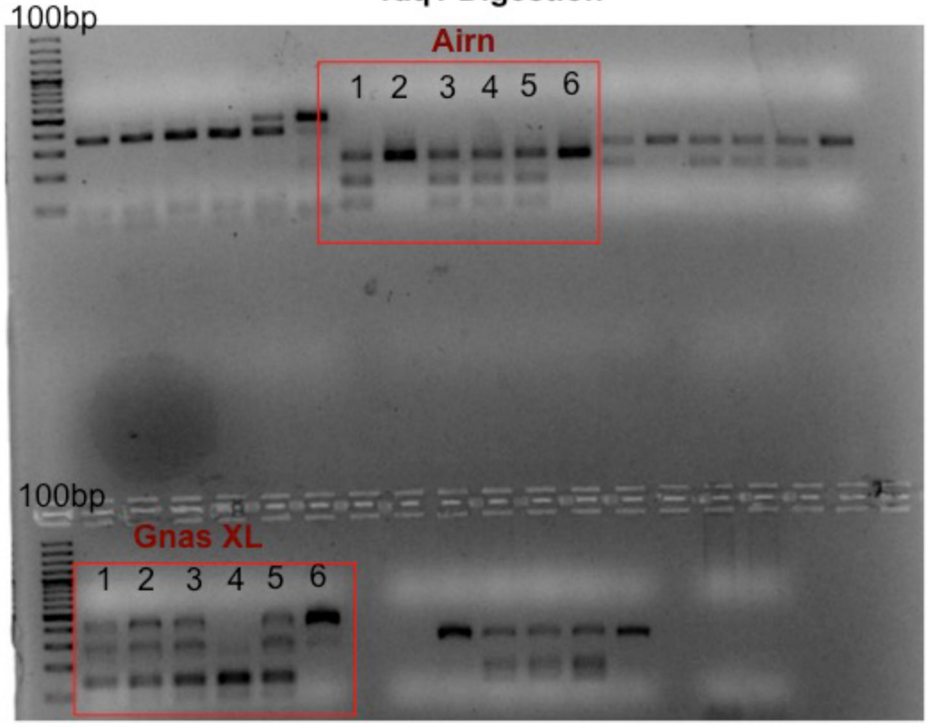

BSTU1 Digestion

1- E14 Y-mESC
2- E14 O-mESC
3- S33Y #1_O
4- S33Y #2_O
5 – Ctrl gDNA
6 –- Zfp57 KO

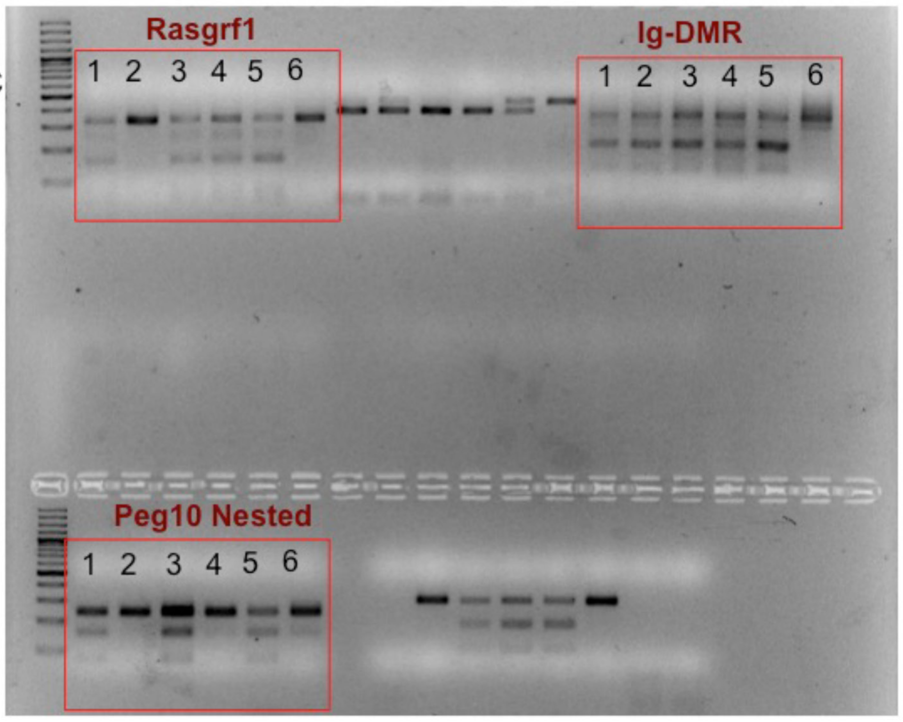

Taq1 **Bstu1**

1- E14 Y-mESC
2- E14 O-mESC
3- S33Y #1_O
4- S33Y #2_O
5 – Ctrl gDNA
6 –- Zfp57 KO
7- E14 Y-mESC bis

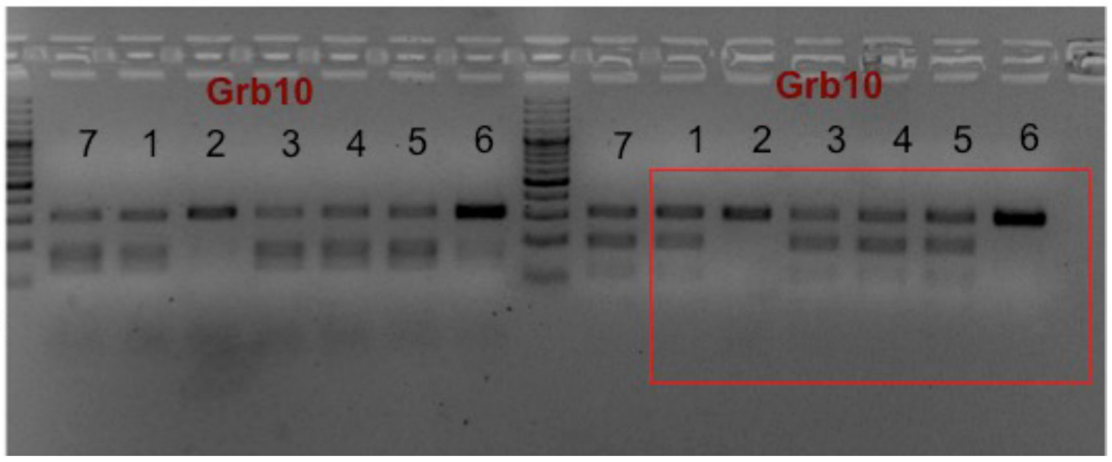

Referring to Fig. S5c

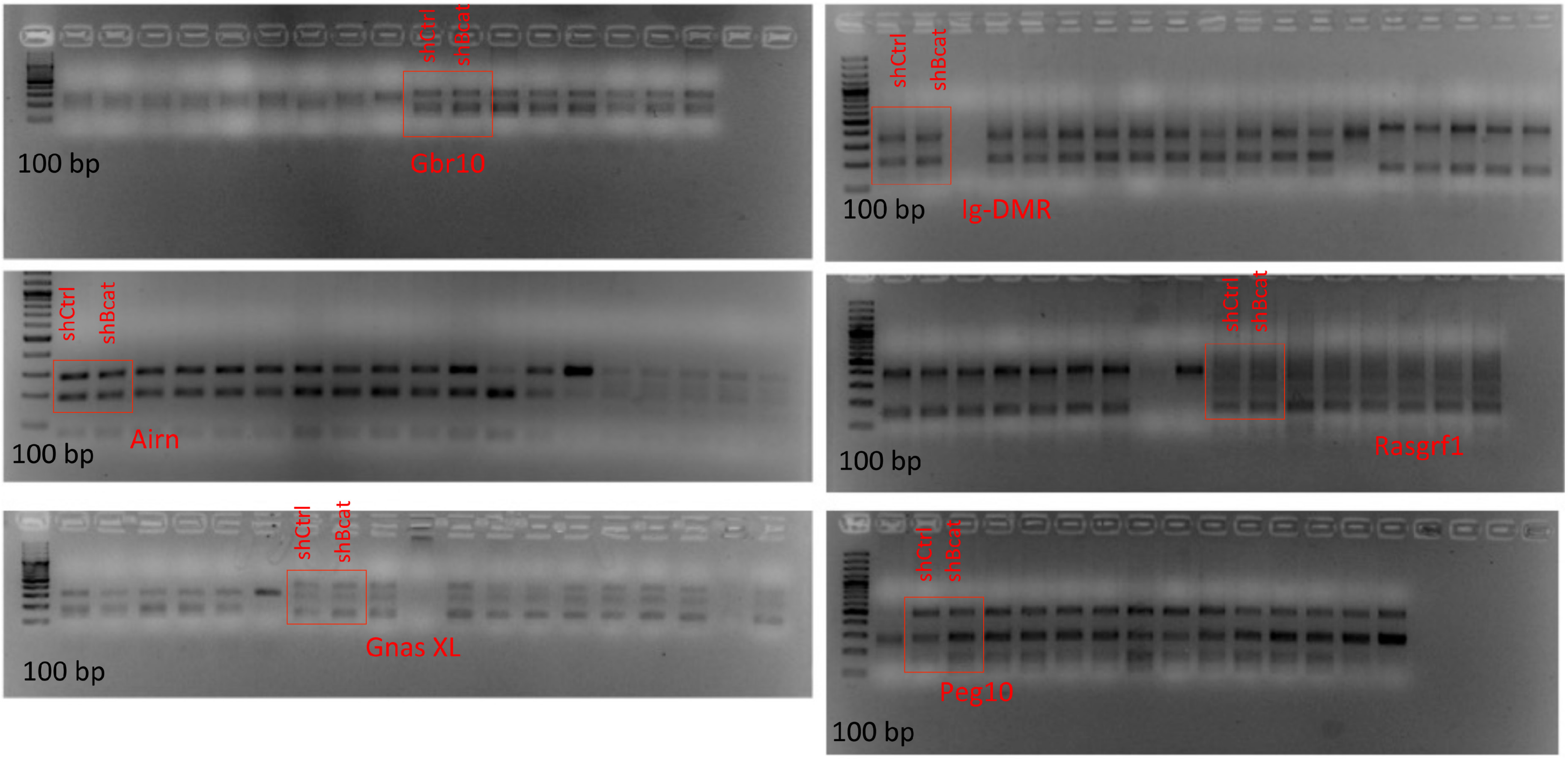

Supplement: Supplementary file 1 — Supplementary Information and figures [file 41598_2018_37442_MOESM1_ESM.pdf]
